# A whale optimization algorithm based on atom-like structure differential evolution for solving engineering design problems

**DOI:** 10.1038/s41598-023-51135-8

**Published:** 2024-01-08

**Authors:** Junjie Tang, Lianguo Wang

**Affiliations:** College of Information Science and Technology, Gansu Agricultural University, No. 1 Yingmen Village, Lanzhou, 730070 Gansu China

**Keywords:** Computational science, Computer science, Mathematics and computing, Mechanical engineering

## Abstract

The whale optimization algorithm has received much attention since its introduction due to its outstanding performance. However, like other algorithms, the whale optimization algorithm still suffers from some classical problems. To address the issues of slow convergence, low optimization precision, and susceptibility to local convergence in the whale optimization algorithm (WOA). Defining the optimization behavior of whale individuals as quantum mechanical behavior, a whale optimization algorithm based on atom-like structure differential evolution (WOAAD) is proposed. Enhancing the spiral update mechanism by introducing a sine strategy guided by the electron orbital center. Improving the random-walk foraging mechanism by applying mutation operations to both the electron orbital center and random individuals. Performing crossover operations between the newly generated individuals from the improved mechanisms and random dimensions, followed by a selection process to retain superior individuals. This accelerates algorithm convergence, enhances optimization precision, and prevents the algorithm from falling into local convergence. Finally, implementing a scouting bee strategy, where whale individuals progressively increase the number of optimization failures within a limited parameter *L*. When a threshold is reached, random initialization is carried out to enhance population diversity. Conducting simulation experiments to compare the improved algorithm with the whale optimization algorithm, other optimization algorithms, and other enhanced whale optimization algorithms. The experimental results indicate that the improved algorithm significantly accelerates convergence, enhances optimization precision, and prevents the algorithm from falling into local convergence. Applying the improved algorithm to five engineering design problems, the experimental results demonstrate that the improved algorithm exhibits good applicability.

## Introduction

The Optimization Problem (OP)^1^ as defined by refers to the task of identifying the optimal choice among various strategies and parameters under specific conditions. This problem is prevalent in real-world applications and encompasses a wide range of scenarios where the goal is to find the best solution within a set of alternatives. Some of the classic intelligent optimization algorithms, including Particle Swarm Optimization (PSO) inspired by bird foraging behaviors^2,3^, Genetic Algorithm (GA) simulating genetic and evolutionary processes^4^, Ant Colony Optimization (ACO)^5^ mimicking ant collective pathfinding, and Simulated Annealing (SA)^6^ emulating material annealing, have been widely applied in various fields. In recent years, researchers have introduced novel intelligent optimization algorithms for solving optimization problems. For instance, the Bat Algorithm (BA)^7^ is inspired by the echolocation behavior of bats in detecting prey and navigating around obstacles. The Grey Wolf Optimization Algorithm (GWO)^8^ draws inspiration from the leadership and hunting behavior of wolf packs. The Hybrid Frog-Leaping Algorithm (SFAL)^9^ is inspired by the foraging mechanisms of frogs in constrained environments. Additionally, the Moth Flame Optimization Algorithm (MFO)^10^ is based on the spiral flight behavior of moths around flames. These emerging algorithms have shown promise in addressing a wide range of optimization challenges. Different intelligent optimization algorithms continue to drive advancements and transformations in the industrial sector and real-world applications. For instance, scheduling problems^11–13^, industrial manufacturing^14,15^, aviation^16,17^, facial recognition^18–20^, and medical imaging^21,22^, among others, have all seen the influence and application of various intelligent optimization algorithms.

The Whale Optimization Algorithm (WOA)^23^ is a novel intelligent optimization algorithm proposed by Australian researchers in 2016. It is inspired by the collective hunting behavior of whales in the natural world. This algorithm offers advantages such as simplicity in principles, fewer parameters, and ease of implementation. It has successfully been applied to solve a variety of problems in fields such as image retrieval^24^, image segmentation^25^, medicine^26^, energy^27^, neural networks^28^, feature selection^29^, wind speed prediction^30^, key recognition^31^, and sentiment analysis^32^, among others. However, WOA still faces challenges when applied to nonlinear, high-dimensional, and complex optimization problems, including issues related to low optimization precision, slow convergence, and susceptibility to local convergence. To address these challenges, researchers have proposed various strategies to enhance WOA.

Improvements to WOA primarily fall into two categories: (1) enhancing WOA through improvements in initialization, parameter settings, and algorithm structure; and (2) leveraging the complementary strengths of WOA with other algorithms.

Parameter tuning in optimization algorithms has a significant impact. Therefore, Chen et al.^33^ introduced an Enhanced Whale Optimization Algorithm with Dual Adaptive Random Alternates. They introduced a random alternate strategy to preserve the positions of better dimensions and incorporated dual adaptive factors from the particle swarm algorithm. The improved algorithm was applied to engineering design problems, and experimental results demonstrated its superior performance compared to other algorithms. Wen et al.^34^ proposed an enhanced Whale Optimization Algorithm for solving large-scale optimization problems. They used an opposition-based learning strategy for population initialization and designed a nonlinear convergence factor. Experiments were conducted on large-scale high-dimensional functions, and the results showed that it outperformed other comparative algorithms. Wang et al.^35^ introduced a Whale Optimization Algorithm based on chaotic search strategy. They employed a chaos reverse learning strategy for population initialization and designed a nonlinear convergence factor along with an inertia weight factor. Performance testing was conducted on 10 benchmark functions and 6 composite functions, with experimental results demonstrating significant improvements in the algorithm’s performance over the baseline. Jiang et al.^36^ introduced an enhanced Whale Optimization Algorithm based on military planning and strategic adjustment. They modified key parameters of the original algorithm to enable classification search. The improved algorithm’s performance was tested using CEC2014 functions and three constrained optimization engineering problems, showing favorable results for optimization tasks. Wen et al.^37^ proposed a whale optimization algorithm based on refraction learning strategy, employed for solving high-dimensional optimization problems and photovoltaic model parameter estimation. They utilized the Logistic model and refraction learning strategy in the improved algorithm and applied it to solve high-dimensional optimization problems, two engineering design problems, and the photovoltaic model parameter estimation problem. Comparative analysis against other algorithms demonstrated its robust performance.

While the Whale Optimization Algorithm (WOA) introduced by Mirjalili and Lewis^23^ exhibits strong performance in solving function optimization problems compared to algorithms like Glover and Marti^5,38–40^, its simplistic algorithm structure still falls short in addressing complex optimization problems. Therefore, improving algorithm structures can effectively enhance algorithm performance. Zhao et al.^41^ proposed an Orthogonal Learning Design Whale Optimization Algorithm with a clustering mechanism. It employs a cluster-based mechanism for population exchange, guiding individuals towards dominant regions in the search space. Experimental results demonstrated the significant effectiveness of the improved algorithm. Agrawal et al.^29,42^ introduced a Quantum Whale Optimization Algorithm for feature selection problems. It represents population individuals using quantum bits and incorporates mutation operators, improved mutations, and crossover operators. Statistical tests showed that the improved algorithm outperforms other metaheuristic algorithms. Liu^43^ proposed a Multi-Population Bidirectional Learning and Information Exchange Whale Optimization Algorithm. This algorithm divides the population into multiple mutually independent subgroups and introduces a linearly decreasing probability of individual replacement to facilitate information exchange between different subgroups. It achieved excellent results in various optimization problems. Jingsen et al.^44^ presented an improved Whale Optimization Algorithm for engineering design optimization problems. They introduced a feedback mechanism based on the current global optimum in the random walk foraging strategy, segment-wise random inertia weight in other strategies, and improved boundary handling. The improved algorithm was applied to 12 complex benchmark test functions and 3 engineering optimization design problems, demonstrating significant performance. Wu and Fei^45^ introduced a Whale Optimization Algorithm based on an improved spiral updating position model. It incorporated opposition learning strategies, random parameter adjustments, and normal mutation operations to enhance the algorithm. Finally, it conducted large-dimensional comparative experiments with high-dimensional functions, comparing IMWOA^45^ with IWOA^34^ and found that IMWOA outperforms the other algorithms.

On the other hand, researchers have aimed to enhance the performance of WOA^23^ by complementing it with other algorithms and have achieved promising results when applying improved versions of the algorithm to real-world problems. For example, Yanfeng et al.^46^ proposed an Enhanced Whale Optimization Algorithm based on the encirclement mechanism. This algorithm utilizes the Tent chaotic map, nonlinear parameters, restricted fitness control, and Gaussian detection mechanisms. It also incorporates the encirclement mechanism from the Harris’s Hawk Algorithm^47^. Experimental results demonstrated significant improvements in convergence precision and speed. Andi et al.^48^ introduced a Chaos-based Multi-Elite Whale Optimization Algorithm, which employs the cubic mapping chaotic operator, incorporates the sine–cosine algorithm^39^, and utilizes multi-elite search strategies. The improved algorithm was validated through testing on 20 benchmark functions and trajectory planning simulations, showing notable enhancements in optimization performance. In response to the global COVID-19 pandemic in 2019, Abdel-Basset et al.^49^ proposed an enhanced Whale Optimization Algorithm combined with the slime mold algorithm for detecting COVID-19 in X-ray images. The algorithm demonstrated significant performance in addressing the challenges posed by COVID-19 chest X-ray images in the proposed metrics.

The aforementioned improved algorithms exhibit better performance compared to the basic Whale Optimization Algorithm. However, they still face some inherent challenges, such as convergence speed, optimization precision, the ability to escape local convergence, and the need for further enhancement in solving engineering design problems, as well as their overall applicability. Therefore, to address the issues of slow convergence, low optimization precision, and susceptibility to local convergence in the Whale Optimization Algorithm, we redefine the optimization behavior of whale individuals as atom-like behavior and propose a whale optimization algorithm based on atom-like structure differential evolution (WOAAD). We define the global optimum individual as the nucleus center and the concentric circle formed by the nucleus center as the electron orbit. We calculate the local optimum individual within the electron orbit and define it as the electron orbit center. In the spiral update mechanism, we introduce a sine-based strategy guided by the electron orbit center, which is combined with the original update process.

In the contraction enclosure mechanism, we retain the nucleus center for approaching prey. In the random walk foraging mechanism, we perform mutation operations on both the electron orbit center and random individuals. This prevents the random individuals from blindly searching. The newly obtained individuals are subjected to crossover operations with random dimensions, followed by a selection process to retain the better individuals. This accelerates the optimization process, improves optimization precision, and helps avoid getting trapped in local optima. Finally, we execute the scout bee strategy, where whale individuals gradually increase the number of optimization failures within the restricted parameter *L*. Once it reaches a threshold, random initialization is performed to enhance population diversity. We conducted simulation experiments comparing the improved algorithm with other algorithms. The results demonstrate a significant improvement in optimization speed and the ability to avoid getting trapped in local optima. We applied the improved algorithm to five engineering design problems: cantilever beam, tension spring, three-bar truss, pressure vessel, and gearbox. The experimental results indicate that the improved algorithm exhibits good applicability in these cases.

The main contributions of this paper include:

1. Introducing a novel version of the Whale Optimization Algorithm (WOAAD) that retains the basic algorithm structure while innovatively incorporating quantum mechanics theory and Bohr atomic model theory. It also introduces a differential evolution mechanism to accelerate algorithm convergence, improve convergence precision, and avoid local optima. Additionally, the scout bee strategy is introduced to enhance population diversity.
2. Demonstrating the significant competitiveness of the proposed algorithm through experiments on 23 standard benchmark functions. Further validation of the algorithm's effectiveness and applicability is provided by solving five mechanical optimization design problems.

The remaining sections of this paper are organized as follows:

Section “Whale Optimization Algorithm” provides an overview of the basic WOA. Section “Whale Optimization Algorithm based on atom-like structure differential evolution” presents the content of the WOAAD algorithm. Section “Time complexity analysis of WOAAD” analyzes the time complexity of the WOAAD algorithm. Section "Convergence analysis of WOAAD" discusses the convergence analysis of the WOAAD algorithm. Section "The experiments and comparisons" presents comparative experiments on standard functions, along with their analysis and discussion. Section "Engineering design problems" discusses experiments related to solving engineering design problems, along with their analysis and discussion. Section “Conclusions” summarizes the conclusions of this paper and suggests directions for future work.

## Whale Optimization Algorithm

**Figure 1:**
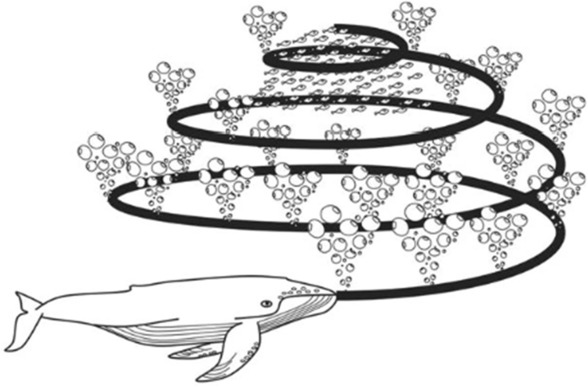
Schematic diagram of bubble net feeding for humpback whales.

The behavior of whales cooperating in hunting, known as bubble-net feeding, is depicted in Fig. 1. During this predation process, whales move in circular or “9”-shaped patterns and release unique bubbles to accomplish their hunting. Researchers have delved deeper into this behavior and found that it can be simulated to solve optimization problems, involving processes such as collective searching, encircling, and pursuit within a whale group.

Assuming a whale population size of N and a search space of D dimensions, the position of the *i*-th whale individual is represented as *X*_*i*_ = (*X*_*i,1*_, *X*_*i,2*_, …, *X*_*i, D*_), where *i* ∈ 1, 2, …, *N*. The current best position within the population is considered the prey. Whale individuals move towards the target prey for encircling, while other individuals in the population move towards the best individual for encirclement. This is achieved by updating their positions using Eq. (1):

1 $$D = |C \cdot X^{*} (t) - X(t)|$$

2 $$X(t + 1) = X^{*} (t) - A \cdot D$$

where, *t* is Current iteration number; *X*(t) is Current individual’s position vector; X* is Position of the prey. The coefficient vectors A and C are defined as follows:

3 $$A = 2a \cdot r_{1} - a$$

4 $$C = 2 \cdot r_{2}$$

where, *r*_1_ and *r*_2_ is random numbers in the range [0, 1]; a is Convergence factor, which linearly decreases from 2 to 0 with an increasing number of iterations.

5 $$a = 2 - \frac{2t}{{t_{\max } }}$$

where, *t* is Current iteration number; *T* is Maximum number of iterations.

In the Whale Optimization Algorithm, there are two different methods: the Contraction Boundary Mechanism and the Spiral Updating Position method. The Contraction Boundary Mechanism is implemented as the convergence factor ‘a’ decreases. In the Spiral Updating Position method, it simulates the spiral behavior of whales, and its mathematical model is as follows.

6 $$X(t + 1) = D \cdot e^{bl} \cdot \cos (2\pi l) + X^{*} (t)$$

where, D =| X*(t)−X(t)| represents the distance between the whale and its prey, where b is a constant used to define the logarithmic spiral shape, and *l* is a random number within the range [− 1, 1]. Here, the whales move around the prey’s shrinking circle while following a spiral path. To simulate this behavior, in the optimization process of the algorithm, the probabilities of choosing the shrinking enclosure mechanism and updating the spiral position are both set to 0.5. Of course, in addition to these strategies, whales can also engage in random foraging. The mathematical model for the random searching behavior of whale individuals can be represented as.

7 $$D = \left| {C \cdot X_{rand} (t) - X(t)} \right|$$

8 $$X(t + 1) = X_{rand} (t) - A \cdot D$$

where *X*_*rand*_(t) represents the position vector of a randomly selected whale individual from the whale population.

In summary, the pseudocode for the Whale Optimization Algorithm (WOA) is presented in Algorithm 1.

**Figure:**
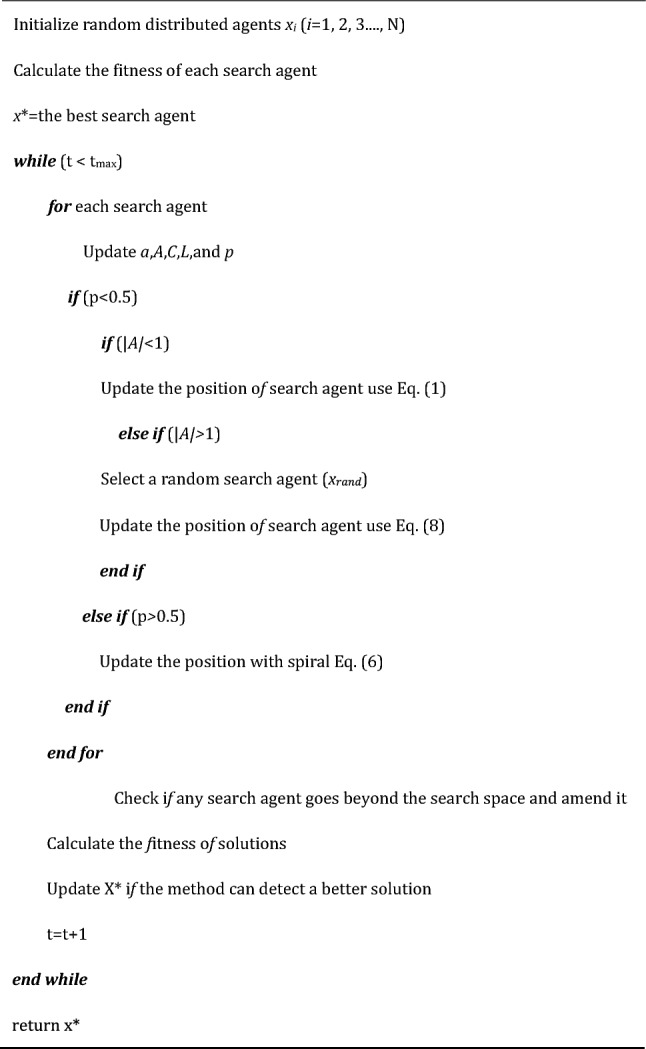
Algorithm 1. Pseudocode of WOA

## Whale Optimization Algorithm based on atom-like structure differential evolution

Quantum mechanics (QM) is a branch of physics that serves as the theoretical foundation for understanding the behavior of microscopic particles in the physical world. It primarily deals with the study of atoms, molecules, condensed matter, as well as the structure and properties of atomic nuclei and fundamental particles.

In this paper, we conceptualize a whale population as a representation of the motion of microscopic particles. Furthermore, we introduce the Bohr atomic model theory^50,51^ into the algorithm. The theory has been intensively applied to optimization problems^52–56^.

In this context, the global best individual and local best individuals are defined as the nucleus center and electron orbit center, respectively. These definitions are utilized in the context of differential evolution to enhance the algorithm’s performance. The mechanisms of the shrinkage and encircling of the whale population, random foraging, and spiral updating are defined as quantum mechanical behaviors. These quantum-inspired behaviors are incorporated into the whale optimization algorithm to propose a novel optimization approach known as the Whale Optimization Algorithm based on Atom-like Structure Differential Evolution (WOAAD).

### Fundamental concepts

#### Definition 1

(Electron Orbit): The search space of the initialized whale population is defined as a quantum space. In this quantum space, there exists an atomic model. Multiple whales can be considered as multiple electrons within this atomic model, with one representing the nucleus center represented by the global best individual.

The current whale individual *X*_*i*_ = (*X*_*i,1*_, *X*_*i,2*_, …, *X*_*i, D*_), where *i* ∈ 1, 2, …, *N*, is defined as the reference position. The group of individuals {*i*,* i* + 1,* i* + 2, …, *i* + *k*-1} is defined as the electron orbit. In the quantum space, there are N initial whale individuals and k electron orbit individuals.

#### Definition 2

(Electron Orbit Center): The local best individual within the electron orbit k is defined as the electron orbit center, denoted as $X^{k} (t) = (X_{1}^{k} ,X_{2}^{k} ,...,X_{D}^{k} )$.

#### Definition 3

(Nucleus Center): The global best individual within the whale population is defined as the nucleus center, denoted as $X^{g} (t) = (X_{1}^{g} ,X_{2}^{g} ,...,X_{D}^{g} )$.

#### Definition 4

(Spiral Updating Mechanism): The position distance parameter in the sine function update formula within the spiral updating mechanism is defined as the vector difference between the electron orbit center and the current individual's position, denoted as $D_{k} = \left| {C \cdot X^{k} - X(t)} \right|$.

#### Definition 5

(Random Foraging Mechanism): The position distance parameter in the random foraging mechanism is defined as the vector difference between the electron orbit center and the position of a randomly selected individual, denoted as $D_{kr} = \left| {C \cdot X^{k} - X_{rand} } \right|$.

### Differential evolution strategy

The Differential Evolution Algorithm (DE)^57^ consists of mutation, crossover, and selection operations. It controls the direction of population individuals by adjusting parameters such as scaling factor and crossover probability. After initializing the population in the algorithm, an individual is randomly chosen as a differential vector. Depending on different mutation strategies, this individual is subjected to mutation operations to generate new individuals. Subsequently, the new individuals are randomly recombined with components from various dimensions to create crossover individuals. Finally, a greedy selection process is employed to retain the better individuals. Here are some commonly used mutation strategies.

9 $${\rm DE}/{\rm rand}/1:\;\;v(t + 1) = x_{r1} + F \cdot (x_{r2} - x_{r3} )$$

10 $${\rm DE}/{\rm best}/1:v(t + 1) = x^{g} + F \cdot (x_{r2} - x_{r3} )$$

11 $${\rm DE}/{\rm current - to - best}/1:v(t + 1) = x_{i} + F \cdot (x^{g} - x_{i} ) + F \cdot (x_{r1} - x_{r2} )$$

12 $${\rm DE}/{\rm best}/2:v(t + 1) = x^{g} + F \cdot (x_{r1} - x_{r2} ) + F \cdot (x_{r3} - x_{r4} )$$

13 $${\rm DE}/{\rm rand}/1:v(t + 1) = x_{r1} + F \cdot (x_{r2} - x_{r3} ) + F \cdot (x_{r4} - x_{r5} )$$

where *v*(t + 1) represents the mutated individual, *F* is the scaling factor, *x*_*g*_ is the global best individual, and *x*_*r*1_, *x*_*r*2_, *x*_*r*3_, *x*_*r*4_, and *x*_*r*5_ are randomly selected individuals.

#### Mutation operations

In the Differential Evolution algorithm, the mutation operation involves generating new mutated individuals *v*(*t*) = (*v*_*i*1_, *v*_*i*2_, *v*_*i*3_, …, *v*_*iN*_) from different individuals in the *t*-th iteration. In this paper, the spiral update mechanism and random foraging mechanism from the WOA are improved using different strategies:

Drawing inspiration from the sine cosine algorithm^39^, the algorithm’s performance is enhanced by utilizing the periodic oscillations of sine and cosine functions. The sine function is introduced into the spiral update mechanism, guided by the electron orbit center. The cosine function guided by the atomic nucleus center is used along with the sine function guided by the electron orbit center with a probability based on a random value *r* = *rand* () for coordinated optimization.

14 $$\begin{gathered} v(t + 1) = D \cdot e^{bl} \cdot \cos (2\pi l) + X^{g} \, r < 0.5 \hfill \\ v(t + 1) = D_{k} \cdot e^{bl} \cdot \sin (2\pi l) + X^{k} \, r > 0.5 \hfill \\ \end{gathered}$$

The random foraging mechanism in the WOA algorithm exhibits pseudo-randomness, where random whales from the population are selected for position updates, adding uncertainty to the algorithm and wasting computational resources. The improved random foraging mechanism determines the new position jointly with the electron orbit center and a randomly selected individual from the population. This approach avoids the blind search of random individuals, effectively enhancing algorithm stability.

15 $$v(t + 1) = X(t) + A \cdot \left| {C \cdot D_{k \, r} } \right|$$

In these mutation operations, the random individual is randomly selected from the current population and may not be equal to the individual being mutated. These different strategies work together and complement each other, effectively balancing the algorithm’s global search and local exploitation capabilities, thus preventing the algorithm from getting stuck in local optima. Additionally, the new individual *v*(t) obtained from the contraction–expansion mechanism, as per Eq. (1), directly proceeds to the subsequent operations.

#### Crossover operation

In the Differential Evolution algorithm, the crossover operation involves generating a crossover individual *u*(*t*) = (*u*_*i1*_, *u*_*i2*_, *u*_*iN*_) by randomly recombining the various components of the mutated individual *v*(*t*) = (*v*_*i1*_, *v*_*i2*_, *v*_*iN*_) and *X*_*i*_. This process enhances population diversity. The components of *u*(*t*) are obtained as follows.

16 $$u(t + 1) = \left\{ {_{{v(t)\quad \, r_{3} \, \ge \, CR \, or \, j \, \ne \, j_{rand} }}^{{v(t) \quad\, r_{3} \, < \, CR \, or \, j \, \ne \, j_{rand} }} } \right.$$

where, *CR* is a constant crossover probability, *r*_3_ is a random number in the range [0, 1], and *j*_*rand*_ ∈ {1, 2, *N*} is a randomly selected dimension index. It ensures that *u*(*t*) must obtain at least one element from *v*(t), ensuring the creation of a new individual.

#### Selection operation

In the Differential Evolution algorithm, the selection strategy involves choosing the superior individual for the next generation based on the fitness values of *X*(*t*) and the crossover individual *u*(*t*). For minimization problems, the selection operation is determined by the following equation.

17 $$X(t + 1) = \left\{ {_{X(t) \, \quad f(u(t)) \, \ge \, f(X(t))}^{u(t) \,\quad f(u(t)) \, < \, f(X(t))} } \right.$$

### Scout Bee strategy

After several iterations, it is possible for individuals to remain unaltered over multiple iterations. Ineffective searching in known areas not only wastes computational resources but may also lead to getting stuck in local optima. The Artificial Bee Colony algorithm^58^, inspired by the characteristics of bee colonies, is a population-based optimization algorithm known for its strong global optimization capabilities, minimal parameters, high precision, and robustness. In this paper, we adopt the scout bee strategy from the Artificial Bee Colony algorithm and design a dynamic expression (Eq. 18). When an individual has remained unchanged for a certain number of consecutive iterations, exceeding the threshold *L* without finding a better solution, it indicates that the individual is trapped in a local optimum. In this case, the individual is randomly initialized, effectively enhancing the diversity of the population and improving global exploration capabilities.

18 $$L = \left\lfloor {t_{\max } /50} \right\rfloor$$

In summary, the pseudocode for the Whale Optimization Algorithm Based on Quantum Centered Differential Evolution (WOAAD) is presented in Algorithm 2.

**Figure:**
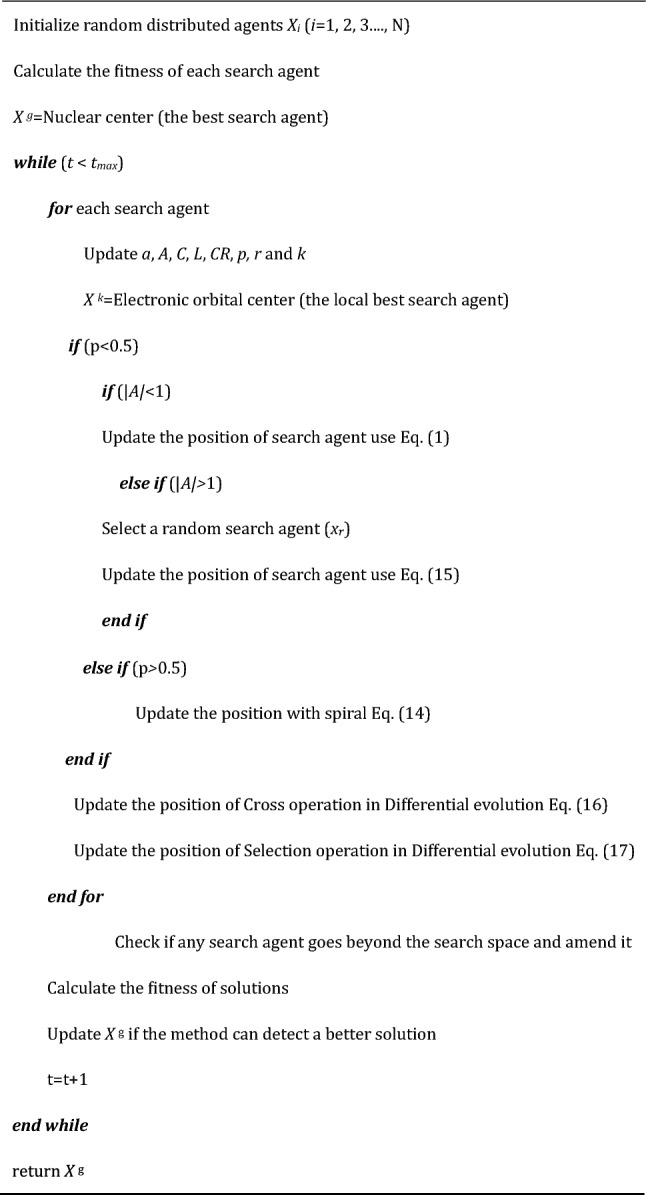
Algorithm 2. Pseudocode of WOAAD

## Time complexity analysis of WOAAD

In intelligent optimization algorithms, time complexity is a significant criterion for evaluating algorithm efficiency. In WOA, assuming a population size of *N* and an individual dimensionality of *n*, the computation of global best individual information is represented by *s*_0_, while *s*_1_ signifies the time required to initialize individuals in each dimension during the algorithm's initialization process. Additionally, *f*(*n*) denotes the time spent on evaluating the fitness function. Therefore, the time complexity associated with the initialization phase in WOA can be expressed as follows.

19 $$O(s_{0} + N(n \cdot s_{1} )) = O(n + f(n))$$

During the iterations of the algorithm, with a maximum number of iterations represented as *t*_*max*_, and assuming that the computation time for other algorithm parameters is *s*_2_, and the time for handling the boundaries of each dimension for individuals is *s*_3_, the time complexity for the parameter setting part can be represented as follows.

20 $$O(N(n \cdot s_{3} + s_{2} ) = O(n + f(n))$$

In WOA, different strategies are executed with certain probabilities. Specifically, the spiral updating strategy is executed with a probability of *p*, while the other strategies are executed with a probability of 1−*p*. Additionally, the parameter *A* plays a crucial role in determining which strategy to execute. Based on these probabilities, we can deduce that there will be *N*_1_, *N*_2_, and *N*_3_ individuals executing three different mechanisms, with dimensionality update times represented as *s*_4_, *s*_5_, and *s*_6_, respectively. Therefore, the time complexity associated with the strategy optimization phase can be expressed as.

21 $$\begin{gathered} O(N(N_{1} (n \cdot s_{4} ) + N_{2} (n \cdot s_{5} ) + N_{3} (n \cdot s_{6} ) \hfill \\ \;\;\; = O(n + f(n)) \hfill \\ \end{gathered}$$

Therefore, the time complexity of WOA is given by the sum of the time complexities for the initialization, parameter settings, and strategy optimization phases.

22 $$\begin{aligned} O & (WOA) \\ & = O(s_{0} + N(n \cdot s_{1} )) + t_{\max } (O(N(n \cdot s_{3} + s_{2} ) \\ & \;\;\; + O(N(N_{1} (n \cdot s_{4} ) + N_{2} (n \cdot s_{5} ) + N_{3} (n \cdot s_{6} )) \\ & = O(n + f(n)) \\ \end{aligned}$$

In comparison to WOA, the improved algorithm WOAAD has the same basic information in the initialization phase, such as population size and individual dimensions. In WOAAD, it is assumed that the individual information for the electron orbital center is represented by *c*_1_. The introduced DE strategy includes the setting of *CR*, which is considered as a constant value and is not included in the calculation. Therefore, the time complexity of the initialization part for WOAAD can be expressed as:

23 $$O(s_{0} + c_{1} + N(n \cdot s_{1} )) = O(n + f(n))$$

During the algorithm's iterative process, the maximum number of iterations, denoted as *t*_*max*_, is the same as in WOA. Additionally, the calculation time for the parameter *L* in the scout bee strategy is represented as *c*_2_. Therefore, the time complexity of the algorithm's parameter setting phase can be expressed as follows:

24 $$O(N(n \cdot s_{3} + s_{2} + c_{2} ) = O(n + f(n))$$

In WOAAD, the fundamental optimization strategy is the same as in WOA. Furthermore, the random walk foraging mechanism utilizes a search strategy based on the vector between the electron orbit center and the current individual's position. The time consumed by this part has already been calculated in the previous sections. However, what differs is the addition of the spiral update mechanism, which includes a sinusoidal function search strategy guided by the electron orbit center. Assuming that this part involves *N*_4_′ individuals and requires *s*_7_′ time for dimension updates, the time complexity of the strategy optimization phase can be expressed as:

25 $$\begin{aligned} O & (N(N_{1} (n \cdot s_{4} ) + N_{2} (n \cdot s_{5} ) + N_{3} (n \cdot s_{6} ) + N_{4} (n \cdot s_{7} ) \\ & = O(n + f(n)) \\ \end{aligned}$$

Due to the introduction of the DE strategy in WOAAD, individuals are required to undergo crossover and selection operations. Assuming that the time required for crossover operation is *w*_1_ and the time required for selection operation is *w*_2_, the time complexity of this part can be expressed as:

26 $$O(N(N_{1} (n \cdot w_{1} ) + N_{2} (n \cdot w_{2} ) = O(n + f(j))$$

Based on the calculations and considerations mentioned above, the time complexity of WOAAD can be summarized as follows:

27 $$\begin{aligned} O & (WOAAD) \\ & = O(s_{0} + c_{1} + N(n \cdot s_{1} )) + t_{\max } (O(N(n \cdot s_{3} + s_{2} + c_{2} ) \\ & \;\;\; + O(N(N_{1} (n \cdot s_{4} ) + N_{2} (n \cdot s_{5} ) + N_{3} (n \cdot s_{6} ) + N_{4} (n \cdot s_{7} )) \\ & = O(n + f(n)) \\ \end{aligned}$$

## Convergence analysis of WOAAD

WOAAD is a stochastic search algorithm, and its global convergence can be analyzed using the general convergence criteria^59^. It is considered to satisfy global convergence if the following two assumptions are met:

### Assumption 1.

28 $$f(E(x,\xi )) \le f(x)\;\;and\; \, if\;\;\xi \in S,f(E(x,\xi )) \le \;\;\min \;\;\{ f(x),f(\xi )\}$$

In Hypothesis 1, denoted by Eq. (28), various symbols and conditions are introduced. Let's clarify their meanings: *E* represents the algorithm under consideration; *S* is defined as the search space, encompassing the set of feasible solutions; *f* stands for the fitness function, the objective function to be optimized by algorithm E; We set *k* = 0, indicating the initial iteration count. For *k* iterations, we use *x*_*k*_, and the subsequent iteration is expressed as *x*_*k*_ + 1 = *P* (*x*_*k*_, *ζ*), where *ζ* represents the solutions discovered during the algorithm E process; Satisfying these conditions allows us to conclude that is monotonically non-increasing. We can formally define it as the lower extreme value of the Lebesgue measure volume.

29 $$\alpha = \inf (t:v\left[ {x \in S|f(x) < t} \right] > 0)$$

In the equation, *v*[*X*] represents the Lebesgue measure on the set *X*, signifying the existence of non-empty subsets within the search space, where the fitness values of members can approach *β* infinitely closely. Consequently, we can define the optimality region as follows:

30 $$R\varepsilon ,M = \left\{ {_{{\left\{ {x \in S|(x) < C\} ,\quad \, \alpha } \right. = \, - \, \infty }}^{{\left\{ {x \in S|(x) < \alpha + \varepsilon \} , \, \quad\alpha \, is} \right. \, finite}} } \right.$$

In the equation, *ε* > 0, and *C* < 0. If a random search algorithm finds a point within *R* ε, *M*, it can be considered that the algorithm has discovered the global optimum or an approximate global optimum.

### Assumption 2.

For any Borel subset A in S with *v*(*A*) > 0, then:

31 $$\mathop \prod \limits_{k = 0}^{\infty } (1 - \mu_{k} (A)) = 0$$

where *μ*_*k*_ is the probability measure of algorithm E on set *L* at step *k*

### Lemma 1.

If the function *f* is measurable, the search space *S* is a measurable subset of *Rn*, and algorithm E satisfies Assumption 1 and Assumption 2, then for the sequence generated by algorithm E, denoted as $\{ x_{k} \}_{k = 0}^{\infty }$, the following holds:

32 $$\mathop {\lim }\limits_{k \to \infty } E(x_{k} \in R\varepsilon ,M) = 1$$

In the equation above, $E(x_{k} \in R\varepsilon ,M)$ represents the probability measure of the point *x*_*k*_ generated by algorithm E at step *k* in the set $R\varepsilon ,M$.

### Theorem 1.

As per Hypothesis 1, it is evident that either condition $x_{k}$ or condition $\xi_{k}$ from $R\varepsilon ,M$ implies condition $x_{k}^{*} \in R\varepsilon ,M$. Hence, all instances of $k^{*} \ge k + 1$ follow.

### Proof.

33 $$E \left(x^{k} \in R\varepsilon ,M \right) = 1 - E \left(x^{k} \in S\backslash R\varepsilon ,M \right) \ge 1 - \mathop \prod \limits_{l = 0}^{K - 1} (1 - u_{l} (R\varepsilon ,M))$$

### Theorem 2.

Algorithm E satisfies condition 2.

34 $$1 \ge \mathop {\lim }\limits_{k \to \infty } E(x_{k} \in R\varepsilon ,M) \ge 1 - \mathop {\lim }\limits_{k \to \infty } \mathop \prod \limits_{l = 0}^{K - 1} (1 - u_{l} (R\varepsilon ,M)) = 1$$

### Theorem 3.

In the WOA algorithm, the iterations ensure that the best individual is smoothly transferred to the next generation through different optimization strategies. Therefore, based on the description, the WOAAD algorithm can be defined as *P*, and the WOAAD algorithm satisfies condition 1, which is expressed as

35 $$P(G_{t} ,K_{t} ,X_{i,t} ) = \left\{ \begin{gathered} G_{t} f(m(X_{i,t} )) \ge f(G_{t} ); \hfill \\ m(X_{i,t} ),f(m(X_{i,t} )) < f(G_{t} ); \hfill \\ \end{gathered} \right.$$

In the equation, the function *m* corresponds to the mutation operation in the differential evolution mechanism. *m* (*Xi*, *t*) represents the updated position of whale individual *i* after the mutation operation at the *t*-th update.

36 $$P(G_{t} ,K_{t} ,X_{i,t} ) = \left\{ \begin{gathered} G_{t} f(c(X_{i,t} )) \ge f(G_{t} ); \hfill \\ c(X_{i,t} ),f(c(X_{i,t} )) < f(G_{t} ); \hfill \\ \end{gathered} \right.$$

In the equation, the function *c* corresponds to the crossover operation in the differential evolution mechanism. *c* (*Xi*, *t*) represents the updated position of whale individual *i* after the crossover operation at the *t*-th update.

37 $$P(G_{t} ,K_{t} ,X_{i,t} ) = \left\{ \begin{gathered} G_{t} f(s(X_{i,t} )) \ge f(G_{t} ); \hfill \\ s(X_{i,t} ),f(s(X_{i,t} )) < f(G_{t} ); \hfill \\ \end{gathered} \right.$$

In the equation, the function *s* corresponds to the selection operation in the differential evolution mechanism. *s* (*Xi*, *t*) represents the updated position of whale individual *i* after the selection operation at the *t*-th update.

*Gt* represents the position of the atomic nucleus center, which is the current global optimum solution, and *Kt* represents the position of the electron orbit center, which is the current local optimum solution. As defined in the previous text, it is known that the fitness values corresponding to *Gt* and *Kt* are non-increasing and gradually converge to the lower bound of the solution space.

### Theorem 4.

The WOAAD algorithm satisfies Hypothesis 2.

### Proof.

The sample space of the whale population *N* must encompass *S*, which can be expressed as:

38 $$S \subseteq \mathop \cup \limits_{i = 1}^{N} M_{i,t}$$

where, *M*_*i*,*t*_ represents the support set of the sample space for individual *i* in the *t*-th generation.

For the optimization mechanism in WOAAD algorithm, after the *t*-th iteration, there exists a positive integer *t*′, such that when *t* > *t*′, the expression for the *j*-th dimension of the *i*-th individual, as well as the probability density function, is given by:

$$P(x_{i,t}^{{}} ) = \prod\limits_{d = 1}^{D} {\frac{1}{{m_{i,t}^{{}} }}\exp \left( {\frac{{ - 2\left| {x_{i,t}^{{}} - (G_{i,t}^{{}} + K_{i,j} )} \right|}}{{m_{i,t}^{{}} }}} \right)\frac{1}{{c_{i,t}^{{}} }}\exp \left( {\frac{{ - 2\left| {x_{i,t}^{{}} - (G_{i,t}^{{}} + K_{i,j} )} \right|}}{{c_{i,t}^{{}} }}} \right)\frac{1}{{s_{i,t}^{{}} }}\exp \left( {\frac{{ - 2\left| {x_{i,t}^{{}} - (G_{i,t}^{{}} + K_{i,j} )} \right|}}{{s_{i,t}^{{}} }}} \right)}$$

39 $$P(x_{i,t}^{{}} ) = \prod\limits_{d = 1}^{D} {\frac{1}{{m_{i,t}^{{}} }}\exp \left( {\frac{{ - 2\left| {x_{i,t}^{{}} - (G_{i,t}^{{}} + K_{i,j} )} \right|}}{{m_{i,t}^{{}} }}} \right)\frac{1}{{c_{i,t}^{{}} }}\exp \left( {\frac{{ - 2\left| {x_{i,t}^{{}} - (G_{i,t}^{{}} + K_{i,j} )} \right|}}{{c_{i,t}^{{}} }}} \right)\frac{1}{{s_{i,t}^{{}} }}\exp \left( {\frac{{ - 2\left| {x_{i,t}^{{}} - (G_{i,t}^{{}} + K_{i,j} )} \right|}}{{s_{i,t}^{{}} }}} \right)}$$

When Borel subsets *P* of *S* satisfy *v*(*P*) > 0, we can obtain the following.

40 $$\eta_{i,t} [P] = \int {_{P} } \left[ {\prod\limits_{d = 1}^{D} {\frac{1}{{m_{i,t}^{{}} }}\exp \left( {\frac{{ - 2\left| {x_{i,t}^{{}} - (G_{i,t}^{{}} + K_{i,j} )} \right|}}{{m_{i,t}^{{}} }}} \right),\;\frac{1}{{c_{i,t}^{{}} }}\exp \left( {\frac{{ - 2\left| {x_{i,t}^{{}} - (G_{i,t}^{{}} + K_{i,j} )} \right|}}{{c_{i,t}^{{}} }}} \right),\;\frac{1}{{s_{i,t}^{{}} }}\exp \left( {\frac{{ - 2\left| {x_{i,t}^{{}} - (G_{i,t}^{{}} + K_{i,j} )} \right|}}{{s_{i,t}^{{}} }}} \right)} } \right]$$

In this equation, if $m_{i,j} ,c_{i,j} ,s_{i,j} < \infty$, then we have $0 < \eta_{i,t} [P] < 1$ and $M_{i,t} = R^{D} \supset S$. Here, *M*_*i*,*t*​_ represents the support of *η*_*i*,*t*_ in the sample space and *P* ⊃ *M*_*i*,*t*_. Therefore, we can conclude that the union of supports of all individuals is.

41 $$M_{t} = \cup_{i = 1}^{N} M_{i,t} = R^{D} \supset S$$

In this equation, *M*_*t*_ represents the support of the distribution *μ*. The probability measure of *P* generated by *μ* can be expressed as.

42 $$\eta_{t} [P] = 1 - \prod\limits_{i = 1}^{N} {(1 - \eta_{i,t} [P])}$$

Hence

43 $$V[P] > 0,\eta_{i} (p) = \sum\limits_{i = 1}^{N} {\eta_{i,t} (P) = 1}$$

That is,

44 $$\mathop \prod \limits_{t = 0}^{\infty } (1 - \eta_{t} [P]) = 0$$

Therefore, based on the above analysis, it can be concluded that the WOAAD algorithm satisfies the assumptions of a globally convergent algorithm. Thus, by Lemma 1, it can be asserted that WOAAD possesses global convergence properties.

## The experiments and comparisons

**Figure 2:**
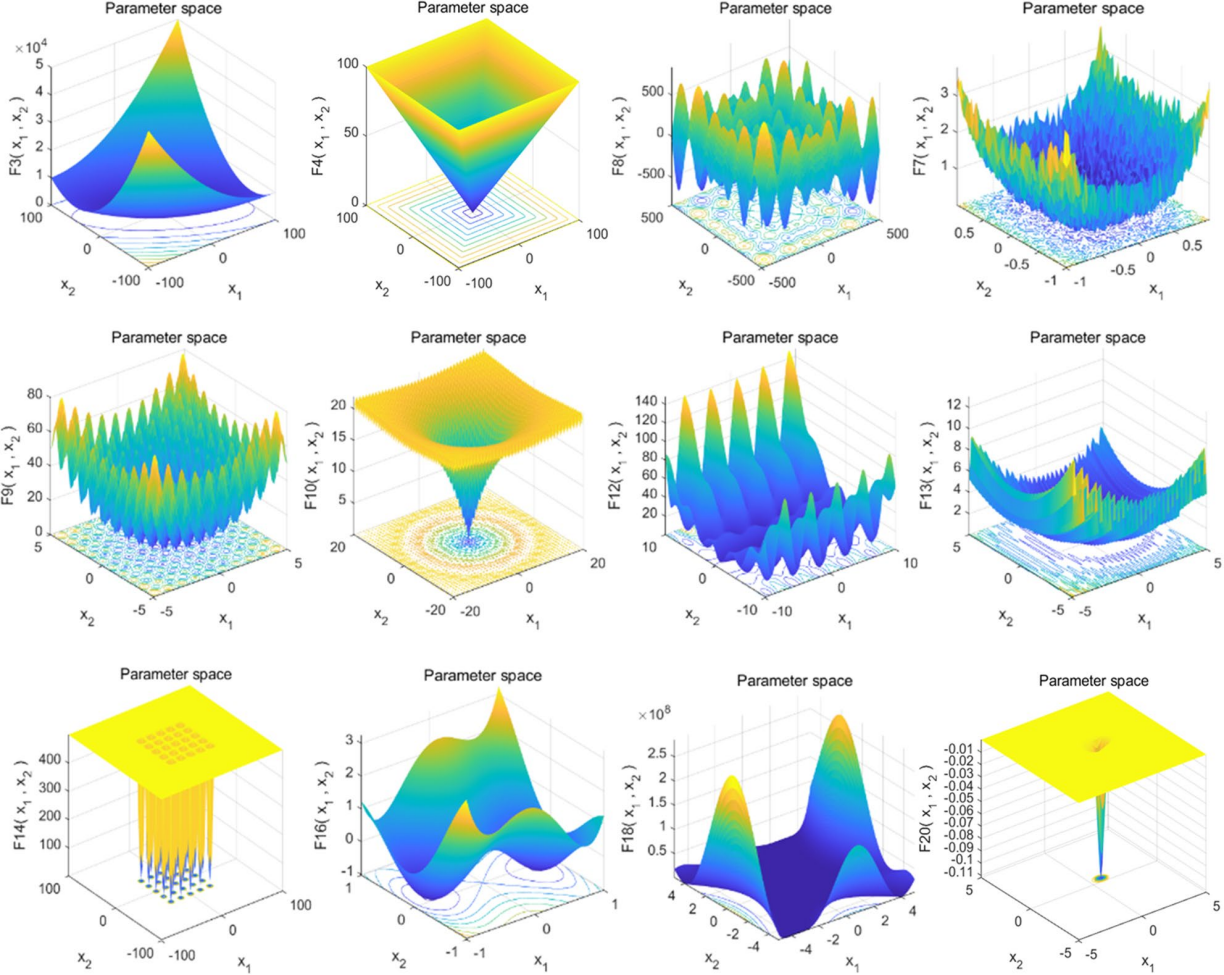
3D graphs of some typical benchmark functions.

To assess the performance of the WOAAD algorithm, this study conducted a series of simulation experiments using 23 standard benchmark functions^60^. These benchmark functions encompass both unimodal functions (f1 to f7) and multimodal functions (f8 to f13), as well as fixed-dimensional multimodal functions (f14 to f23). These functions were selected for their diverse characteristics and varying degrees of difficulty in achieving global optimal solutions. The parameters for these test functions are provided in Table 1. Below are 3D plots depicting some representative standard functions shown in Fig. 2.

**Table 1:** Parameters of 23 standard benchmark functions.

Name	*F*unction	Dim	Range	*F*_min_	Name	*F*unction	Dim	Range	*F*_min_
Sphere	*f*_*1*_	30	[− 100, 100]	0	Penalized2	*f*_*13*_	30	[− 50, 50]	0
Schwe*f*el 2.22	*f*_*2*_	30	[− 10, 10]	0	Shekel *F*oxholes	*f*_*14*_	2	[− 65, 65]	1
Schwe*f*el 1.2	*f*_*3*_	30	[− 100, 100]	0	Kowalik	*f*_*15*_	4	[− 5, 5]	0.00030
Schwe*f*el 2.21	*f*_*4*_	30	[− 100, 100]	0	Six_Hump Camel_Back	*f*_*16*_	2	[− 5, 5]	− 1.0316
Rosenbrock	*f*_*5*_	30	[− 30, 30]	0	Branin	*f*_*17*_	2	[− 5, 5]	0.398
Step	*f*_*6*_	30	[− 100, 100]	0	Goldstein_Price	*f*_*18*_	2	[− 2, 2]	3
QuarticWN	*f*_*7*_	30	[− 1.28, 1.28]	0	Hartman1	*f*_*19*_	3	[0, 1]	− 3.86
Schwe*f*el2.26	*f*_*8*_	30	[− 500, 500]	-12,569.5	Hartman2	*f*_*20*_	6	[0, 1]	− 3.32
Rastrigin	*f*_*9*_	30	[− 5.12, 5.12]	0	Sheke_5	*f*_*21*_	4	[0, 10]	-10.1532
Ackley	*f*_*10*_	30	[− 32, 32]	0	Shekel_7	*f*_*22*_	4	[0, 10]	-10.4028
Griewank	*f*_*11*_	30	[− 600, 600]	0	Shekel_10	*f*_*23*_	4	[0, 10]	-10.5363
Penalized1	*f*_*12*_	30	[− 50, 50]	0

The experimental setup for this study consisted of a system running Windows 10 64-bit on an AMD Ryzen 7 5800H processor with 16 GB of RAM. All experiments were conducted using MATLAB R2019a.

### Evaluation indicators

In this section, four evaluation metrics were utilized to gauge the performance of the algorithm.

1. **Mean.** The average solution obtained from running the algorithm independently 30 times. It is calculated as.
  45 $$Mean = \frac{1}{T}\sum {_{i = 1}^{T} f_{i} }$$
2. **Standard Deviation.** This metric measures the dispersion or spread of fitness values. It is used to assess the level of variability in the dataset and is computed as.
  46 $$SD = \sqrt {\frac{1}{T - 1}\sum {_{i = 1}^{T} (f_{i} - Mean)} }$$
3. **Worst.** The worst fitness value obtained among the independent runs. It indicates the highest fitness value achieved.
4. **Best.** The best fitness value obtained among the independent runs. It represents the lowest fitness value achieved.
  47 $$Worst,best = \left\{ \begin{gathered} \max f_{i} ,1 \le i \le T \hfill \\ \min f_{i} ,1 \le i \le T \hfill \\ \end{gathered} \right.$$
  where, *f*_*i*_ denotes the fitness value for the *i*-th run of the algorithm; *T* is the total number of independent runs of the algorithm.

### Comparison experiment of WOAAD algorithm with other optimization algorithms

In this section, we conducted comparative experiments to evaluate the performance of the WOAAD algorithm against several other optimization algorithms, including the WOA^23^, the Grey Wolf Algorithm^8^, the Harris Hawk Algorithm^47^, and the Salp Swarm Algorithm^61^. The results of these experiments are presented in Table 2. To ensure fair comparisons, all algorithms were configured with uniform parameters: a population size of *N* = 30 and a maximum iteration count of *t*max = 500. The parameter settings for the WOAAD algorithm are as follows: constant *b* = 1, *k* = 5, and *CR* = 0.5. The remaining parameters for the other algorithms were set according to their respective original literature. Each algorithm was independently executed 30 times, and the best results are highlighted in bold in the comparison table.

**Table 2:** The optimization results of WOA, GWO, HHO and WOAAD.

*F*unction	Algorithms	Best	Worst	Mean	SD
*f*_1_	WOAAD	0	0	0	0
	WOA	3.8016e−84	5.5736e−73	2.2179e−74	1.0189e−73
	HHO	4.1359e−116	5.5627e−96	2.3896e−97	1.0261e−96
	GWO	1.2752e−29	9.9795e−27	8.8652e−28	1.7978e−27
	SSA	349.9787	160.1822	585.6567	104.9715
*f*_2_	WOAAD	0	0	0	0
	WOA	1.0723e−59	3.5846e−51	2.304e−52	6.7264e−52
	HHO	1.5047e−58	2.1266e−49	1.1679e−50	4.0863e−50
	GWO	2.1534e−17	3.0324e−16	9.8187e−17	7.1806e−17
	SSA	5.9329	16.0439	11.0885	2.5342
*f*_3_	WOAAD	0	0	0	0
	WOA	30,569.0906	75,655.1687	49,827.4082	12,621.0261
	HHO	6.7466e−100	1.6596e−75	7.0408e−77	3.1101e−76
	GWO	1.5171e−08	0.00036692	1.8288e−05	6.7217e−05
	SSA	1617.5671	10,564.1312	5415.5913	2454.002
*f*_4_	WOAAD	0	0	0	0
	WOA	2.224	88.9313	60.6109	26.3882
	HHO	2.606e−58	2.1574e−48	1.0467e−49	4.1916e−49
	GWO	6.8918e−08	2.162e−06	6.3851e−07	5.3574e−07
	SSA	11.2376	28.9086	19.1829	4.3269
*f*_5_	WOAAD	0.01299	28.7512	24.995	9.6478
	WOA	26.9858	28.818	27.8969	0.36887
	HHO	1.6906e−06	0.087922	0.012376	0.018074
	GWO	26.0538	28.7675	27.2375	0.84853
	SSA	6248.809	73,748.5994	26,561.4827	17,964.0779
*f*_6_	WOAAD	0	1.3538e−12	1.3378e−13	3.4353e−13
	WOA	0.09356	0.76614	0.32394	0.17272
	HHO	1.9586e−08	0.00082251	0.00013159	0.00021025
	GWO	0.24388	1.4806	0.80887	0.37708
	SSA	119.977	580.8091	299.5929	99.5266
*f*_7_	WOAAD	7.7054e−06	0.0016439	0.00021498	0.00036221
	WOA	0.00017931	0.023895	0.0034506	0.0048787
	HHO	4.8214e−06	0.00039979	0.00011996	0.00010308
	GWO	0.00052163	0.0043125	0.0015926	0.0010061
	SSA	0.13346	0.71437	0.29577	0.12572
*f*_8_	WOAAD	− 12,569.4866	− 9212.2855	− 12,351.7831	637.6677
	WOA	− 12,569.4863	− 6538.6952	− 10,308.062	1906.065
	HHO	− 12,569.4865	− 11,858.7627	− 12,537.6845	134.4068
	GWO	− 7776.6086	− 3635.7481	− 6040.2944	905.2374
	SSA	− 8314.9885	− 4503.7983	− 6511.0074	1046.0184
*f*_9_	WOAAD	0	0	0	0
	WOA	0	0	0	0
	HHO	0	0	0	0
	GWO	5.6843e−14	18.1399	3.4543	4.8807
	SSA	86.4546	168.3639	136.1698	22.4511
*f*_10_	WOAAD	8.8818e−16	8.8818e−16	8.8818e−16	0
	WOA	8.8818e−16	7.9936e−15	3.9672e−15	2.5945e−15
	HHO	8.8818e−16	8.8818e−16	8.8818e−16	0
	GWO	7.9048e−14	1.4655e−13	1.1067e−13	1.7288e−14
	SSA	4.4674	7.0992	6.2352	0.73225
*f*_11_	WOAAD	0	0	0	0
	WOA	0	0.20269	0.011016	0.043061
	HHO	0	0	0	0
	GWO	0	0.03333	0.0058945	0.0097911
	SSA	2.2407	5.5809	3.6086	0.8501
*f*_12_	WOAAD	1.5705e−32	4.1559e−13	1.3854e−14	7.5875e−14
	WOA	0.0021747	0.6731	0.053854	0.12374
	HHO	9.1456e−09	0.0001671	1.9895e−05	3.3609e−05
	GWO	0.019602	0.086905	0.0464	0.021015
	SSA	12.0544	50.8527	21.4979	8.227
*f*_13_	WOAAD	1.3498e−32	0.099222	0.0033074	0.018115
	WOA	0.19234	1.061	0.52912	0.21779
	HHO	2.7567e−07	0.00074823	0.00010577	0.0001519
	GWO	0.19569	0.95751	0.5486	0.16911
	SSA	30.763	19,387.8932	1293.6111	3586.6206
*f*_14_	WOAAD	0.998	0.998	0.998	1.6493e−16
	WOA	0.998	10.7632	2.5721	2.7161
	HHO	0.998	5.9288	1.4923	1.2892
	GWO	0.998	12.6705	5.3674	4.5168
	SSA	0.998	2.9821	1.3948	0.80721
*f*_15_	WOAAD	0.0003181	0.0022519	0.00063837	0.00048664
	WOA	0.00030831	0.0015959	0.00060264	0.00034325
	HHO	0.00031158	0.00044182	0.00035629	3.4657e−05
	GWO	0.00030749	0.020363	0.0051599	0.0085359
	SSA	0.00080767	0.0016345	0.0011294	0.00025323
*f*_16_	WOAAD	− 1.0316	− 1.0316	− 1.0316	1.468e−09
	WOA	− 1.0316	− 1.0316	− 1.0316	1.2078e−09
	HHO	− 1.0316	− 1.0316	− 1.0316	1.5069e−09
	GWO	− 1.0316	− 1.0316	− 1.0316	2.5e−08
	SSA	− 1.0316	− 1.0281	− 1.0308	0.00079622
*f*_17_	WOAAD	0.39789	0.39926	0.39795	0.00025266
	WOA	0.39789	0.39831	0.39791	7.6839e−05
	HHO	0.39789	0.39802	0.3979	3.4401e−05
	GWO	0.39789	0.39802	0.39789	2.3516e−05
	SSA	0.39795	0.40621	0.39924	0.001829
*f*_18_	WOAAD	3	3.0015	3.0001	0.00029943
	WOA	3	3.0269	3.0009	0.0048957
	HHO	3	3	3	2.5959e−06
	GWO	3	3.0002	3	4.0305e−05
	SSA	3.0003	3.258	3.0628	0.05742
*f*_19_	WOAAD	− 3.8519	− 2.1001	− 3.3111	0.39229
	WOA	− 3.8628	− 3.8219	− 3.8572	0.010211
	HHO	− 3.8628	− 3.8551	− 3.861	0.0018916
	GWO	− 3.8628	− 3.8549	− 3.8615	0.0025282
	SSA	− 3.8524	− 3.4878	− 3.7302	0.12673
*f*_20_	WOAAD	− 3.2318	− 2.731	− 3.0658	0.11342
	WOA	− 3.3219	− 3.0614	− 3.2483	0.091984
	HHO	− 3.265	− 2.8352	− 3.1231	0.098049
	GWO	− 3.322	− 3.0869	− 3.2955	0.063009
	SSA	− 2.8826	− 1.2784	− 2.5952	0.26936
*f*_21_	WOAAD	− 10.1532	− 10.1532	− 10.1532	5.6943e−15
	WOA	− 10.1519	− 2.6301	− 7.9425	2.7948
	HHO	− 9.9188	− 5.0401	− 5.2147	0.88846
	GWO	− 10.1529	− 2.688	− 9.2276	2.1388
	SSA	− 9.0729	− 2.2544	− 5.4779	2.2054
*f*_22_	WOAAD	− 10.4028	− 10.4028	− 10.4028	3.7686e−12
	WOA	− 10.4025	− 1.8375	− 6.6248	3.1284
	HHO	− 5.0874	− 3.593	− 5.0322	0.27191
	GWO	− 10.4026	− 5.1275	− 10.225	0.96277
	SSA	− 9.844	− 2.2064	− 6.7549	2.2647
*f*_*23*_	WOAAD	− 10.5363	− 10.5363	− 10.5363	9.9068e−15
	WOA	− 10.536	− 2.4215	− 7.8055	3.1418
	HHO	− 10.1667	− 1.6669	− 5.1764	1.1341
	GWO	− 10.5355	− 10.5327	− 10.5344	0.000872
	SSA	− 9.937	− 3.9937	− 7.8785	1.7481

From Table 2, it can be observed that in terms of average best values and standard deviations, the comparison experiments between the WOAAD and the WOA show the following results: For functions f9, f15, f16, and f17, the average best values of WOAAD are comparable to WOA. For functions f15 and f17, the standard deviations of WOAAD are worse than WOA, while for functions f9 and f16, the standard deviations are similar to WOA. For the remaining 19 functions, the optimization results of WOAAD are superior to WOA. In the comparison experiments between the WOAAD and the Harris Hawk Optimization (HHO) algorithm: For functions f9, f10, f11, f16, and f17, the average best values of WOAAD are comparable to HHO. For function f17, the standard deviation of WOAAD is worse than HHO. For functions f9, f10, f11, and f16, the standard deviations are similar to HHO. For functions f7, f8, f13, f15, and f18, both the average best values and standard deviations of WOAAD are worse than HHO. For the remaining 13 functions, the optimization results of WOAAD are superior to HHO. In the comparison experiments between the WOAAD and the Grey Wolf Optimization (GWO) algorithm: For functions f16, f17, and f18, the average best values of WOAAD are comparable to GWO. For functions f19 and f20, the standard deviations of WOAAD are worse than GWO. For the remaining 18 functions, the optimization results of WOAAD are superior to GWO. In the comparison experiments between the WOAAD and the Salp Swarm Algorithm (SSA): For functions f16 and f17, the average best values of WOAAD are comparable to SSA. The standard deviations of WOAAD are better than SSA. For the remaining 21 functions, the optimization results of WOAAD are superior to SSA. Regarding the maximum and minimum values: In the comparison with the WOA, WOAAD shows comparable results for 14 functions and superior results for the remaining 9 functions. In the comparison with HHO, WOAAD demonstrates comparable results for 11 functions and superior results for the remaining 12 functions. In the comparison with GWO, WOAAD achieves comparable results for 10 functions and superior results for the remaining 13 functions. In the comparison with SSA, WOAAD performs comparably for 4 functions and superiorly for the remaining 19 functions.

### Comparison experiments between WOAAD algorithm and other improved WOA algorithms

Next, we compare the WOAAD with other improved whale optimization algorithm (WOA) variants, including enhanced WOA (EWOA)^62^, Improved WOA (IWOA)^63^, and improved WOA with modified spiral update (IMWOA)^45^. The experimental results are shown in Table 3. All compared algorithms are configured with uniform parameters: population size *N* = 30, maximum iterations *t*max = 500, and WOAAD parameters as follows: constant *b* = 1; *CR* = 0.5. To ensure fairness, each compared algorithm is independently run 30 times, and the best values in the comparison results are highlighted.

**Table 3:** The optimization results of WOA, GWO, HHO and WOAAD.

Function	Evaluation criterion	EWOA	IWOA	IMWOA	WOAAD
*f*_1_	Mean	1.3597e−149	3.2291e−119	**0**	**0**
	SD	1.923e−149	4.5638e−119	**0**	**0**
*f*_2_	Mean	7.6987e−83	6.1872e−70	8.82e−181	**0**
	SD	1.3329e−82	1.0542e−69	**0**	**0**
*f*_3_	Mean	1.293e−13	30,539.6732	**0**	**0**
	SD	2.2084e−13	15,486.7023	**0**	**0**
*f*_4_	Mean	6.1656e−48	80.3907	4.27e−184	**0**
	SD	1.0679e−47	7.9271	**0**	**0**
*f*_5_	Mean	28.7989	28.7989	4.29e−05	**24.995**
	SD	27.1782	0.016037	1.33e−04	**9.6478**
*f*_6_	Mean	0.63821	3.0134	**0**	1.3378e−13
	SD	0.33835	0.55587	**0**	3.4353e−13
*f*_7_	Mean	0.0026091	0.0017601	**0**	0.00021498
	SD	0.0022209	0.0022305	**0**	0.00036221
*f*_8_	Mean	− 7593.7712	− 11,563.8375	**− 12,455.60**	**− 12,351.7831**
	SD	1105.5206	1223.8682	**172.0869**	**637.6677**
*f*_9_	Mean	0	0	**0**	**0**
	SD	0	0	**0**	**0**
*f*_10_	Mean	3.2567e−15	3.2567e−15	**8.88e−016**	**8.8818e−16**
	SD	2.0512e−15	2.0512e−15	**1.00e−031**	**0**
*f*_11_	Mean	0	0	**0**	**0**
	SD	0	0	**0**	**0**
*f*_12_	Mean	0.049635	0.31748	1.68e−08	**1.3854e−14**
	SD	0.014566	0.23679	1.70e−08	**7.5875e−14**
*f*_13_	Mean	1.2306	1.7124	**5.69e−06**	0.0033074
	SD	0.51421	0.50525	**1.96e−05**	0.018115
*f*_14_	Mean	1.9881	1.0089	**0.998**	**0.998**
	SD	1.7149	0.0056574	**1.11e−06**	**1.6493e−16**
*f*_15_	Mean	0.00055866	0.0032339	**0.0004**	0.00063837
	SD	0.00016021	0.0033891	**0.000043**	0.00048664
*f*_16_	Mean	-1.0316	− 1.0316	− 1.0316	**− 1.0316**
	SD	2.4912e−10	4.3683e−10	**1.86e−05**	1.468e−09
*f*_17_	Mean	0.39789	0.39789	0.397664	0.39795
	SD	4.3405e−07	2.5836e−06	1.768e−3	0.00025266
*f*_18_	Mean	12.0007	21.9012	**3**	3.0001
	SD	15.5896	16.3696	**1.34e−06**	0.00029943
*f*_19_	Mean	− 3.8611	− 3.8012	− 3.85988	-3.3111
	SD	0.0027449	0.061781	0.00676	0.39229
*f*_20_	Mean	− 3.2758	− 3.0059	− 3.10233	− 3.0658
	SD	0.077888	0.052379	0.084930	0.11342
*f*_21_	Mean	− 8.4535	− 9.0035	− 10.15316	**− 10.1532**
	SD	2.943	1.0662	8.99e−05	**5.6943e−15**
*f*_22_	Mean	− 8.6265	− 7.9479	− 10.40277	**− 10.4028**
	SD	3.0647	2.8226	8.54e−05	**3.7686e−12**
*f*_23_	Mean	− 6.9311	− 5.9503	− 10.53628	**− 10.5363**
	SD	3.1222	3.4744	5.22e−05	**9.9068e−15**

From Table 3, it can be observed that. In single-peak functions f1–f7, WOAAD outperforms both EWOA and IWOA in terms of optimization results. In comparison with IMWOA, WOAAD is worse for functions f6 and f7, equivalent for functions f1 and f3, and superior for the remaining 3 functions. In multi-peak functions f8–f13, WOAAD surpasses EWOA and IWOA. In comparison with IMWOA, WOAAD is worse for function f13, equivalent for functions f8, f9, f10, and f11, and superior for function f12. In fixed-dimension multi-peak functions f14–f23, WOAAD equals EWOA for functions f15, f16, and f17, outperforms EWOA for the remaining 6 functions. In comparison with IWOA, WOAAD equals IWOA for functions f16, f17, and f20, outperforms IWOA for the remaining 6 functions. In comparison with IMWOA, WOAAD equals IMWOA for functions f14, f16, f17, f21, f22, and f23, is worse for functions f15 and f18, and outperforms IMWOA for the remaining 2 functions.

### Convergence curve analysis

**Figure 3:**
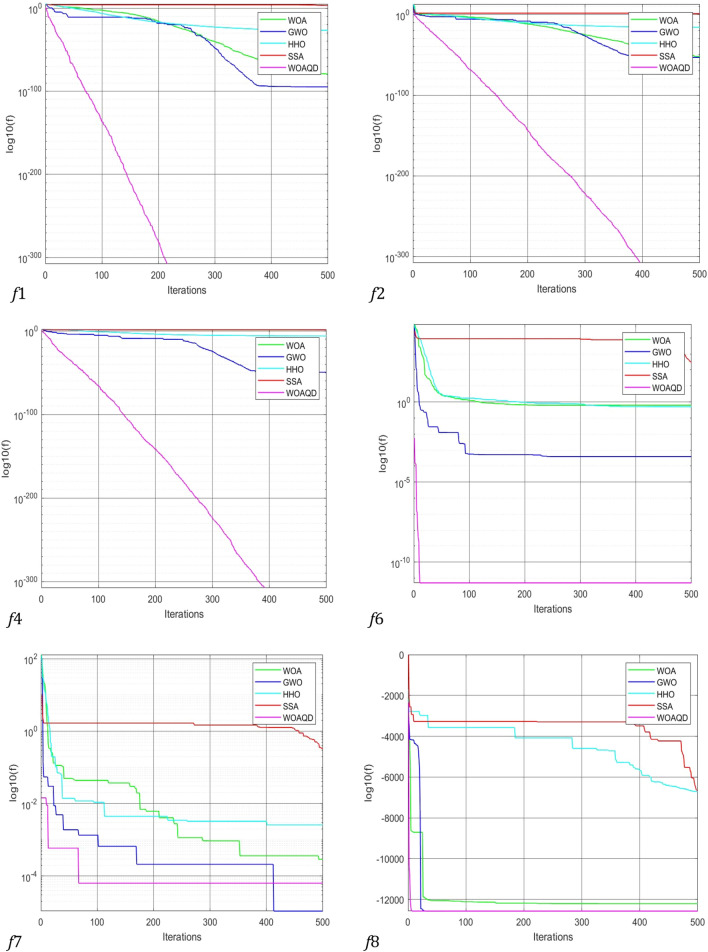

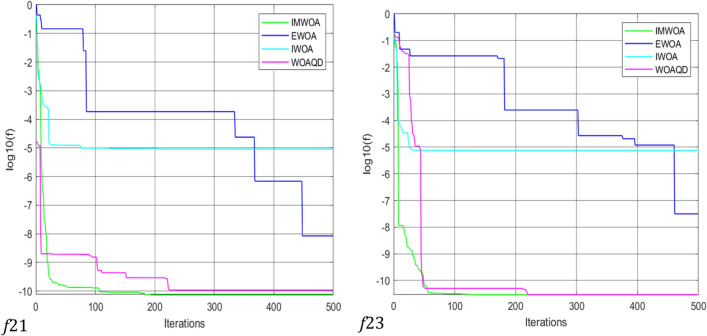
Convergence curve for optimization results of WOA, GWO, HHO and WOAAD.

**Figure 4:**
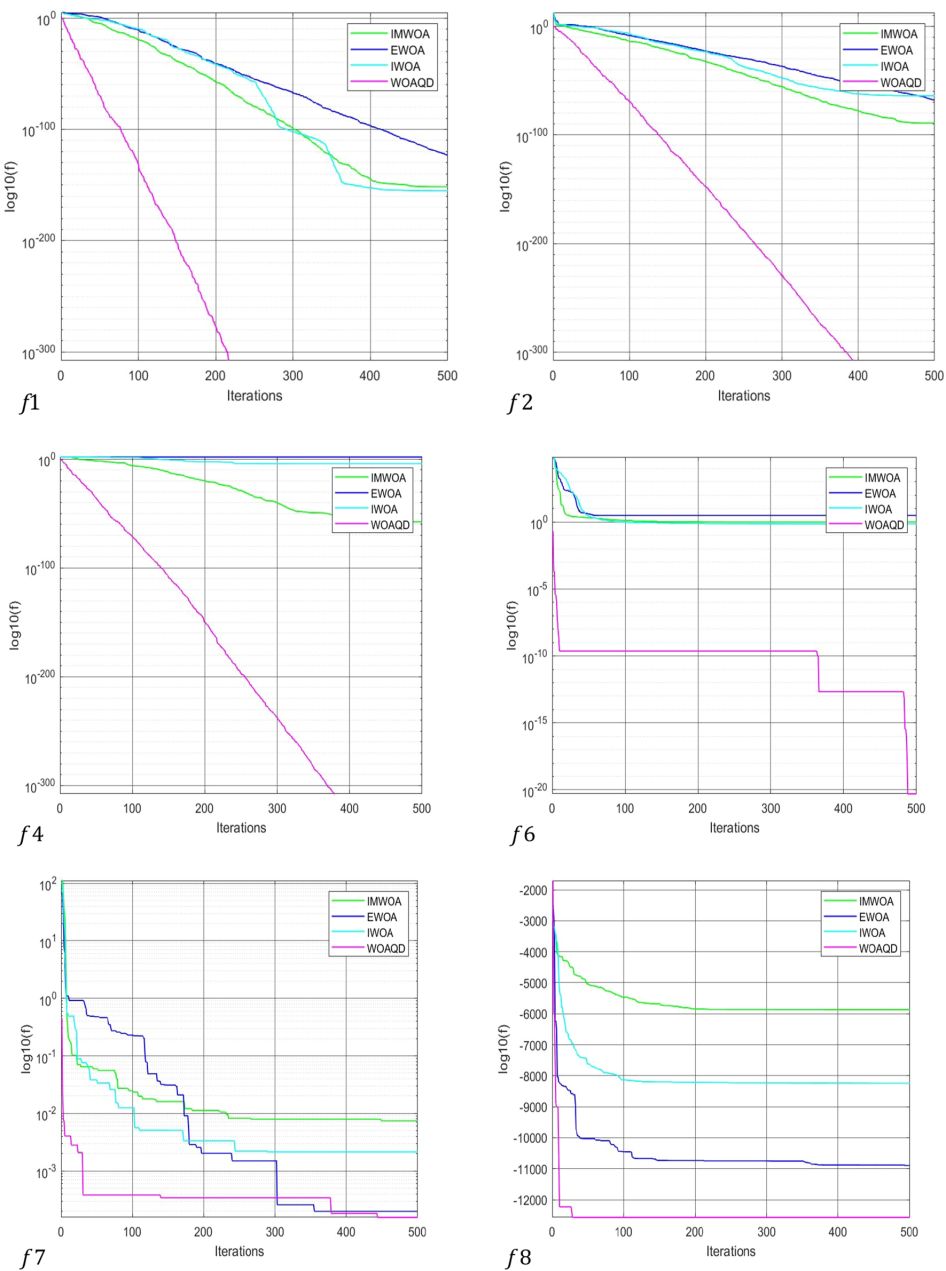

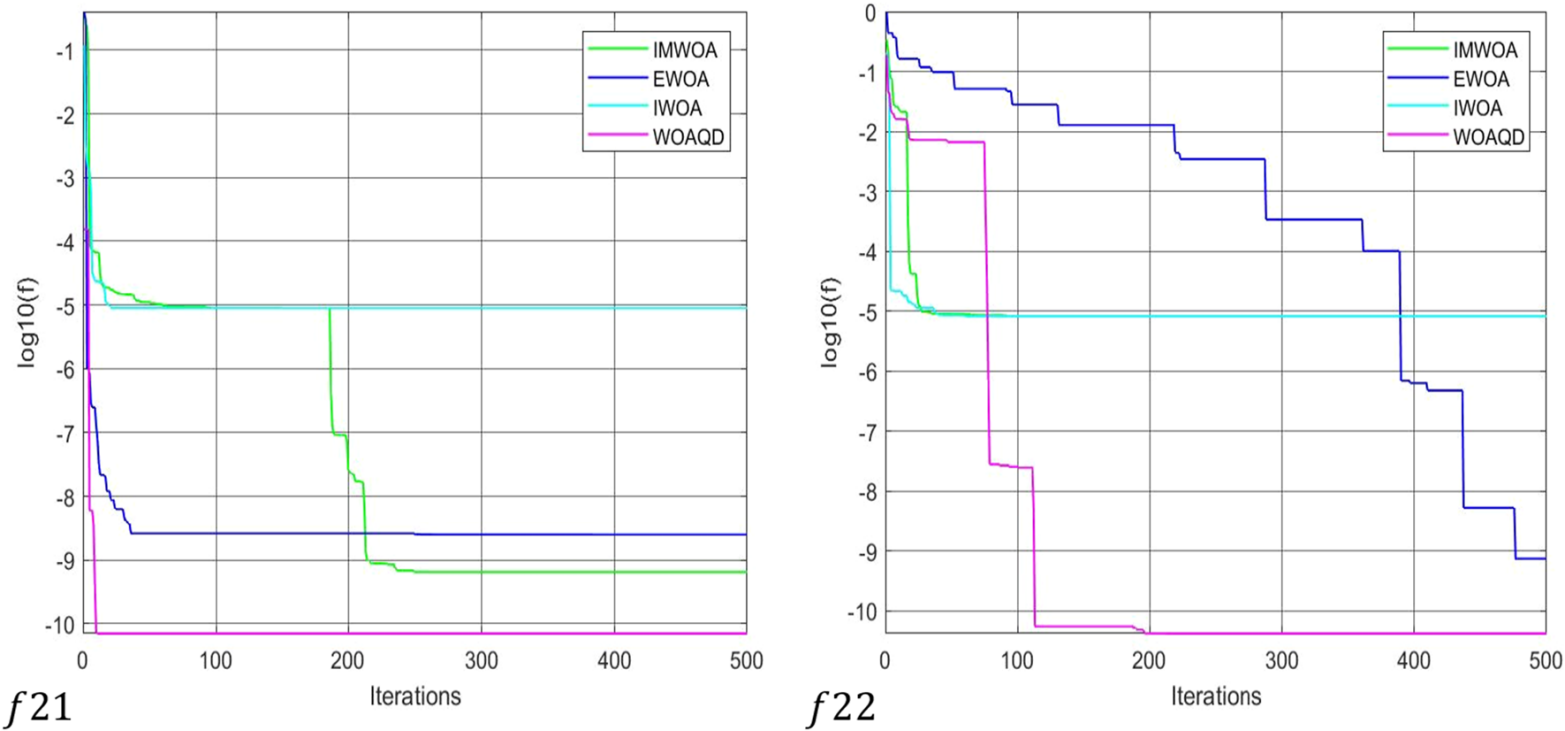
Convergence curve for optimization results of IWOA, EWOA, IMWOA and WOAAD.

To provide a more intuitive view of the convergence performance of the WOAAD, the data from Tables 2 and 3 are plotted as convergence curves. Figures 3 and 4 depict the convergence curves of WOAAD against other optimization algorithms and improved WOA, respectively. From Figs. 3 and 4, it is evident that the WOAAD exhibits superior convergence speed and accuracy compared to the other algorithms under comparison. This is attributed to several factors, including the incorporation of quantum mechanics theory in the WOAAD, the redefinition of leadership in individual optimization using atomic and electronic centers, the introduction of the sine–cosine-based spiral update mechanism, and the integration of strategies led by atomic and electronic centers in the context of the sine–cosine function. These enhancements contribute to the stability of finding the optimal values and increase the success rate of optimization problems. Additionally, the adoption of differential evolution strategies, such as mutation, crossover, selection operations, and scouting bee strategy, accelerates convergence, enhances population diversity, and reduces the likelihood of getting stuck in local optima during the early stages of optimization.

### Analysis and conclusion

In summary, the analysis results indicate that the WOAAD outperforms the other algorithms compared in this study. Specifically, for functions such as f1, f2, f3, f4, f5, f9, and f11, the WOAAD achieves average best values that have already converged to the theoretical optimum. For functions like f8, f14, f16, f18, f21, f22, and f23, the average best values obtained by the WOAAD are very close to the theoretical optimum. Although a small number of functions have not yet converged to the optimal values, overall, the proposed WOAAD demonstrates faster convergence and a higher likelihood of escaping local optima when compared to other optimization algorithms, making it more competitive.

## Engineering design problems

In real-life scenarios, there are numerous optimization problems that are highly non-linear, high-dimensional, and constrained, such as engineering design problems. To validate the feasibility and applicability of the WOAAD, it was applied to traditional engineering design problems. The results of this validation are presented in the following sections.

### Optimization model

The process of formulating mathematical models for engineering design problems involves several steps, including defining design variables, formulating objective functions, and specifying constraints. Engineering design problems^64^ as described by are classic problems within the engineering domain. They are typically represented as constrained optimization problems, and their mathematical models can be expressed as follows:

48 $$\begin{gathered} \, \min f(x) \hfill \\ subject \, to \, g_{p} (x) \le 0,p = 1,2,...,j; \hfill \\ \, h{}_{m}(x) = 0,m = 1,2,...,y; \hfill \\ \, x_{ub} \le x_{i} \le x_{lb} ,i = 1,2,...,n; \hfill \\ \end{gathered}$$

In the above equation, the optimization problem consists of design variables represented by x, where *x* = (*x*_1_, *x*_2_, …, *x*_*n*_) ∈ *R*_*n*_, and *f*(*x*) is the objective function. The constraints include g_*p*_ for the *p*-th inequality constraint and hm for the *m*-th equality constraint. *x*_*ub*_ and *x*_*lb*_ represent the upper and lower bounds of the design variables.

To solve constrained optimization problems, they are typically transformed into unconstrained optimization problems using penalty functions. The penalty function method involves adding a penalty term to the objective function to penalize solutions that do not satisfy the constraints. This transformation allows the constrained optimization problem to be converted into a series of unconstrained subproblems, which can be solved using standard unconstrained optimization methods. The penalty function is expressed as follows.

49 $$F(x) = f(x) + \lambda \left[ {h^{2} (x) + \min \{ 0,g(x)\}^{2} } \right]$$

In the equation, *F*(*x*) represents the penalty function, f(x) is the original objective function of the optimization problem, λ is the penalty factor, *h*2(*x*) is the penalty term related to equality constraints, and min {0, *g*(*x*)}2 is the penalty term related to inequality constraints. The choice of the penalty factor λ has a significant impact on the algorithm. When *λ* is too large, it can lead to premature convergence of the algorithm, making it difficult to search for the optimal solution. When *λ* is too small, it may not achieve the desired penalty effect. The value of *λ* is typically determined through extensive experimentation.

### Experimental parameter settings

To validate the feasibility and applicability of the improved algorithm, it was applied to three engineering design problems: the cantilever beam design problem, the tension spring design problem, and the three-bar truss design problem. These problems were then subjected to simulation experiments and compared against optimization algorithms such as Whale Optimization Algorithm (WOA), Grey Wolf Optimization (GWO), Harris Hawks Optimization (HHO), and Salp Swarm Algorithm (SSA).To ensure the fairness of the experiments, the parameters for each algorithm in the comparative experiments were set as follows: Population size *N* = 30, Maximum number of iterations *T* = 500, Each algorithm was independently run 30 times, and the results were averaged for comparison.

### The Cantilever beam design problem

**Figure 5:**
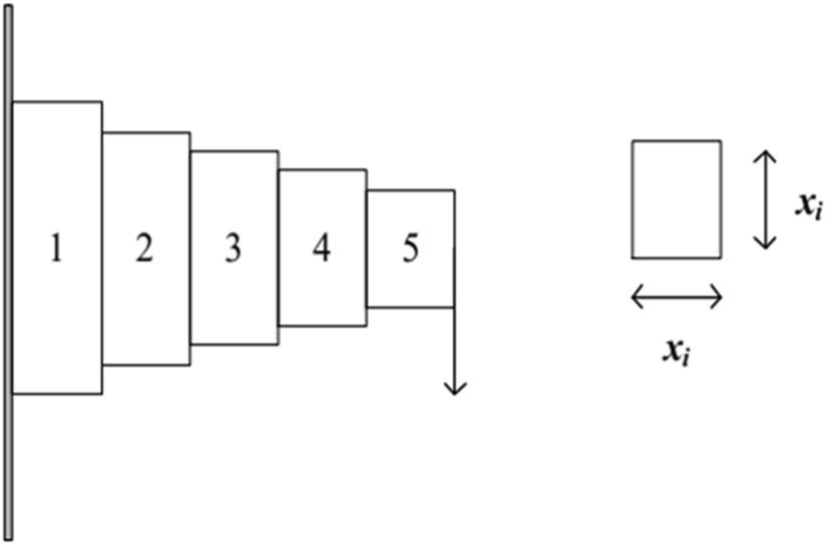
Cantilever beam design problems.

The optimization objective in the cantilever beam design problem^65^ is to minimize the mass of a cantilever beam with a rectangular cross-section. (As in Fig. 5).

The mathematical expression for this problem is as follows.

50 $$\begin{gathered} Funtion: \, \min f(x) = 0.0624\sum\limits_{i = 1}^{5} {x_{i} } \hfill \\ Subject \, to\left\{ \begin{gathered} \, \frac{61}{{x_{1}^{3} }} + \frac{37}{{x_{2}^{3} }} + \frac{19}{{x_{3}^{3} }} + \frac{7}{{x_{4}^{3} }} + \frac{1}{{x_{5}^{3} }} \le 1 \hfill \\ \, 0.01 \le x_{i} \le 100 \, i = 1,2,3,4,5; \hfill \\ \end{gathered} \right. \hfill \\ \end{gathered}$$

where, the function *f*(*x*) represents the minimum value, which corresponds to the optimal mass of the cantilever beam with a rectangular cross-section. The design variables, *x*_*i*_, represent the height or width of different beam segments.

Table 4 presents the performance comparison results of the WOAAD and other optimization algorithms for the cantilever beam design problem. For the minimum value function *f*(*x*), the WOAAD achieved an optimization result of *f*(*x*) = 1.3651, which is significantly better than the results of the WOA and SSA. Although it is on par with the results of the GWO and HHO, it is important to note that the WOAAD also satisfies the constraint conditions for the design variables (*x*_1_, *x*_2_, *x*_3_, *x*_4_, *x*_5_) = (5.4684, 5.3526, 4.5886, 3.6447, 2.8231).

**Table 4:** Performance comparison of different algorithms for cantilever beam design problem.

Algorithms	*x*1	*x*2	* x*3	*x*4	*x*5	*f* (*x*)
WOA	6.7223	5.6496	4.8678	2.7854	1.5343	1.7224
GWO	6.0505	5.3133	4.4703	3.5221	2.1857	1.3402
HHO	6.2829	5.2835	4.4123	3.6826	2.0938	1.3415
SSA	6.7314	4.3729	4.4344	2.9367	4.1988	1.8389
WOAAD	5.4684	5.3526	4.5886	3.6447	2.8231	1.3651

### The tension spring design problem

**Figure 6:**
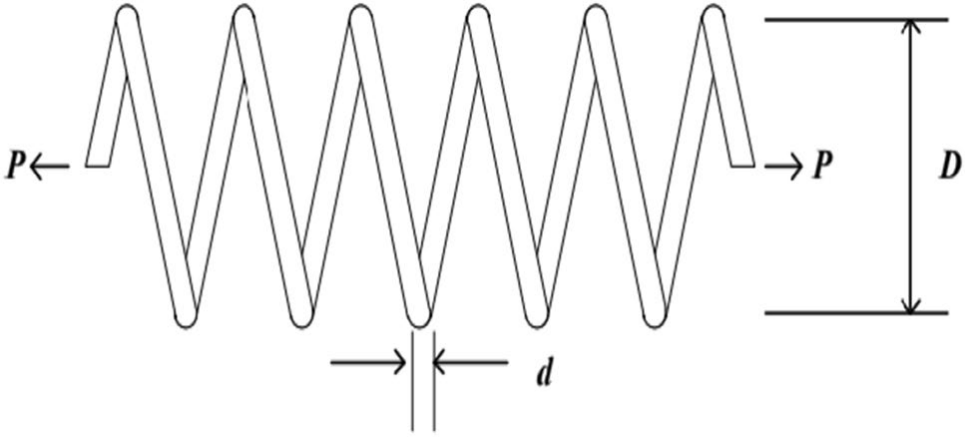
Stretch spring design problems.

The objective of the tension spring design problem^66^ is to minimize the weight of a tension spring while satisfying constraints on coil curvature, shear stress, natural frequency, outer diameter, and restrictions on three design variables. The three design variables are the average coil diameter *D*, wire diameter d, and the effective number of coils *p*. (As in Fig. 6).

The mathematical expression is as follows.

51 $$\begin{gathered} Function: \, \min \, f(x) = (x_{3} + 2)x_{2} x_{1}^{2} \hfill \\ Subject \, to \, \left\{ \begin{gathered} \, g_{1} (x) = 1 - \frac{{x_{2}^{3} x_{3} }}{{71785x_{1}^{4} }} \le 0, \hfill \\ \, g_{2} (x) = \frac{{4x_{2}^{4} - x_{1} x_{2} }}{{12566(x_{2} x_{1}^{3} - x_{1}^{4} )}} + \frac{1}{{5108x_{1}^{2} }} - 1 \le 0, \hfill \\ \, g_{3} (x) = 1 - \frac{{140.45x_{1} }}{{x_{2}^{2} x_{3} }} \le 0, \hfill \\ \, g_{4} (x) = \frac{{x_{1} + x_{2} }}{1.5} - 1 \le 0, \hfill \\ \, 0.05 \le x_{1} \le 2, \hfill \\ \, 0.25 \le x_{2} \le 1.3, \hfill \\ \, 2 \le x_{3} \le 15 \hfill \\ \hfill \\ \end{gathered} \right. \, \hfill \\ \end{gathered}$$

where, the function* f*(*x*) represents the minimum weight of the tension spring, where *d*(*x*_1_) is the wire diameter, *D*(*x*_2_) is the average coil diameter, and *p*(*x*_3_) is the effective number of coils, corresponding to the design variables.

Table 5 presents the performance comparison results of the WOAAD with other optimization algorithms for the tension spring design problem. It is evident that the optimization results of the WOAAD outperform the other optimization algorithms considered in the comparison.

**Table 5:** Performance comparison of different algorithms for stretch spring design problem.

Algorithms	*x*1	* x*2	* x*3	*f* (*x*)
WOA	0.059163	0.56462	4.8865	0.014497
GWO	0.053750	0.55220	5.0087	0.015346
HHO	0.050000	0.31226	14.7351	0.013226
SSA	0.054396	0.39918	9.1854	0.013833
WOAAD	0.053306	0.39688	9.2718	0.012712

### The three-bar truss design problem

**Figure 7:**
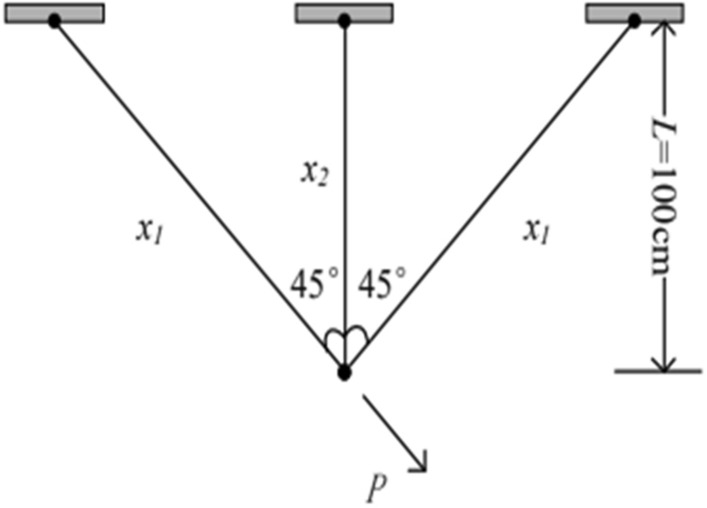
Three-bar truss optimization design problems.

The objective of the three-bar truss design problem^66^ is to find the optimal volume of a three-bar truss by adjusting the cross-sectional areas. This problem involves a nonlinear objective function, three inequality constraints, and two decision variables. (As in Fig. 7).

The mathematical expression is as follows.

52 $$\begin{gathered} Function: \, \min f(x) = (2\sqrt {2x_{1} } + x_{2} ) \cdot l) \hfill \\ Subject \, to\left\{ \begin{gathered} g_{1} (x) = \frac{{\sqrt {2x_{1} } + x_{2} }}{{\sqrt {2x_{1}^{2} + 2x_{1} x_{2} } }}p - \sigma \le 0; \hfill \\ \, g_{2} (x) = \frac{{x_{2} }}{{\sqrt {2x_{1}^{2} + 2x_{1} x_{2} } }}p - \sigma \le 0; \hfill \\ \, g_{3} (x) = \frac{1}{{\sqrt {2x_{1}^{2} + x_{1} } }}p - \sigma \le 0; \hfill \\ \, 0 \le x_{i} \le 1, \, i = 1,2; \hfill \\ l = 100cm,p = 2KN/cm^{2} ,\sigma = 2KN/cm^{2} \hfill \\ \hfill \\ \end{gathered} \right. \hfill \\ \end{gathered}$$

where, the equation, *f*(*x*) represents the objective function, seeking to minimize the optimal volume of the three-bar truss. Variables *l*, *p*, and *σ* represent the deflection, buckling, and stress constraints of the truss components, respectively. *x*_1_​ and *x*_2​_ represent the lengths of the two side truss members, which are evaluated for the best cross-sectional area. The optimization results for the three-bar truss design problem using different algorithms are shown in Table 6.

**Table 6:** Performance comparison of different algorithms for three-pole truss design problem.

Algorithms	Worst	Best	SD	*x*_1_	*x*_2_	*f* (*x*)
WOA	267.7761	263.8988	1.2117	0.7975	0.3837	265.1009
GWO	264.2903	271.0781	2.2715	0.8146	0.3410	267.6625
HHO	263.8972	265.0614	0.2509	0.7879	0.4286	264.0875
SSA	263.8973	263.9679	0.0160	0.7873	0.4121	263.9181
WOAAD	265.0797	264.0048	0.60851	0.80111	0.37417	264.3775

Table 6 presents the performance comparison results of the WOAAD with other optimization algorithms for the three-bar truss design problem. In terms of the objective function's minimum value, the WOAAD obtained an optimization result of *f*(*x*) = 264.3775, which is significantly better than the results obtained by the WOA and SSA. Although it is on par with the results of the GWO and HHO, the WOAAD demonstrates stable performance and satisfies the constraint conditions, as indicated by the other optimization parameters (Worst, Best, standard deviation, *x*_1_, *x*_2_) = (265.0797, 264.0048, 0.60851, 0.80111, 0.37417).

### The pressure vessel design problem

**Figure 8:**
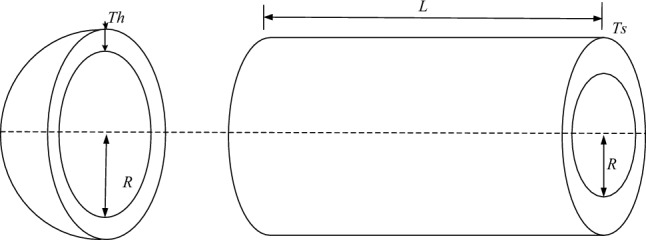
Pressure vessel design problems.

The pressure vessel design problem^67^ aims to minimize the total cost of a pressure vessel, including material costs, manufacturing costs, welding costs, and other constraints. (As in Fig. 8).

The mathematical expression is as follows.

53 $$\begin{gathered} Function: \, \min \, f(x) = 0.6224x_{1} x_{3} x_{4} + 1.7781x_{2} x_{3}^{2} + 3.1661x_{1}^{2} x4 + 19.84x_{1}^{2} x_{3} \hfill \\ Subject \, to \, \left\{ \begin{gathered} \, g_{1} (x) = 0.0193x_{3} - x_{1} \le 0, \hfill \\ \, g_{2} (x) = 0.0095x_{3} - x_{2} \le 0, \hfill \\ \, g_{3} (x) = - \pi x_{3}^{2} x4 - \frac{4}{3}\pi x_{3}^{3} + 1296000 \le 0, \hfill \\ \, g_{4} (x) = x_{4} - 240 \le 0, \hfill \\ \, 0.0625 \le x_{1} \le 6.1875,0.0625 \le x_{2} \le 6.1875,10 \le x_{3} \le 200,10 \le x_{4} \le 200 \hfill \\ \end{gathered} \right. \, \hfill \\ \end{gathered}$$

where, the function *f*(*x*) represents the minimum total cost of the pressure vessel, where TS(× 1) stands for the shell thickness, Th(*x*_2_) represents the head thickness, *R*(*x*_3_) is the inner radius of the head, and *L*(*x*_4_) is the length of the cylindrical cross-section. The design variables *x*_1_ and *x*_2_ must be multiples of 0.0625 inches, while the other two variables are continuous.

Table 7 presents the performance comparison results of the WOAAD and other optimization algorithms for the pressure vessel design problem. Regarding the minimum function value *f*(*x*), the WOAAD achieved an optimization result of *f*(*x*) = 8807.7454, which is significantly suitable for the pressure vessel problem. Moreover, when considering other parameters, the WOAAD demonstrates stable performance and satisfies the constraint conditions.

**Table 7:** Performance comparison of different algorithms for three-pole truss design problem.

Algorithms	*X*_1_	*X*_2_	*X*_3_	*X*_4_	*f* (*x*)
WOA	1.34436	0.570476	58.01	45.1957	7947.518
GWO	0.82375	0.411093	42.611	170.4589	5991.1183
HHO	0.96086	0.481215	49.187	105.8834	6395.352
SSA	1.36397	0.63473	65.356	11.4551	7936.1061
WOAAD	1.38743	0.674322	57.8508	46.1296	8807.7454

### The Gearbox design problem

**Figure 9:**
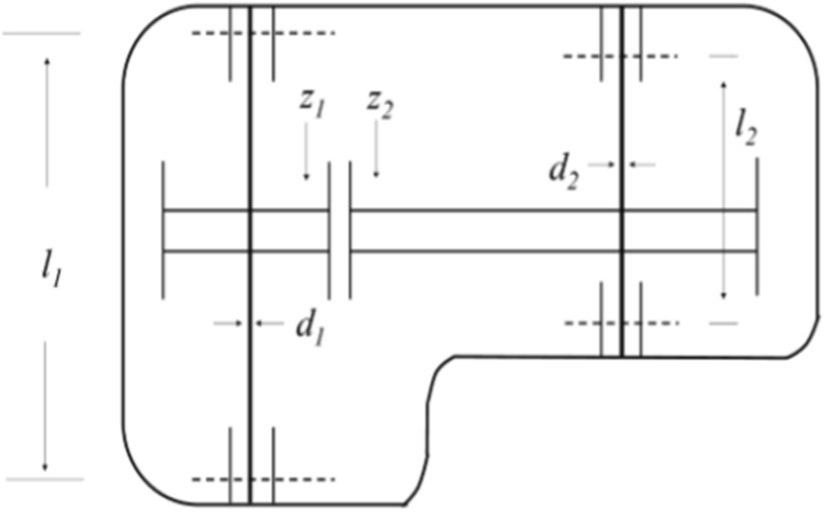
Speed reducer design problem.

The objective of the gearbox design problem^65^ is to minimize the total weight of the gearbox, which includes seven variables: face width *y*_1_, module *y*_2_, number of teeth on the small gear *y*_3_, length of the first shaft between bearings *y*_4_, length of the second shaft between bearings *y*_5_, diameter of the first shaft *y*_6_, and diameter of the second shaft *y*_7_. In this case, there are 11 constraint conditions mainly based on the stress conditions experienced by various components in the system. (As in Fig. 9).

The mathematical expression for this problem is as follows.

54 $$\begin{gathered} Function:\min \, f(x) = 0.7854y_{1} y_{2}^{2} (3.333y_{3}^{2} + 14.9334y_{3} - 43.0934) \hfill \\ - 1.508y_{1} (y_{6}^{2} + x_{7}^{2} ) + 7.4777(x_{6}^{3} + x_{7}^{3} ) + 0.7854(x_{4} x_{6}^{2} + x_{5} x_{7}^{2} ) \hfill \\ Subject \, to \, \left\{ \begin{gathered} \, g_{1} (y) = \frac{27}{{y_{1} y_{2}^{2} y_{3} }} - 1 \le 0, \hfill \\ \, g_{2} (y) = \frac{397.5}{{y_{1} y_{2} y_{3}^{2} }} - 1 \le 0, \hfill \\ \, g_{3} (y) = \frac{{1.9y_{4}^{3} }}{{y_{2} y_{3} y_{6}^{4} }} - 1 \le 0, \hfill \\ \, g_{4} (y) = \frac{{1.9y_{4}^{3} }}{{y_{2} y_{3} y_{6}^{4} }} - 1 \le 0, \hfill \\ \, g_{4} (y) = \frac{{1.93y_{5}^{3} }}{{y_{2} y_{3} y_{7}^{4} }} - 1 \le 0, \hfill \\ \, g_{5} (y) = \frac{1}{{110y_{6}^{3} }}\sqrt {(\frac{{745y_{5} }}{{y_{2} y_{3} }}) + 16.9(10^{6} )} - 1 \le 0, \hfill \\ \, g_{6} (y) = \frac{1}{{85y_{7}^{3} }}\sqrt {(\frac{{745y_{5} }}{{y_{2} y_{3} }}) + 157.5(10^{6} )} - 1 \le 0, \hfill \\ \, g_{7} (y) = \frac{{y_{2} y_{3} }}{40} - 1 \le 0, \hfill \\ \, g_{8} (y) = \frac{{5y_{2} }}{{y_{1} }} - 1 \le 0, \hfill \\ \, g_{9} (y) = \frac{{y_{1} }}{{12y_{2} }} - 1 \le 0, \hfill \\ \, g_{10} (y) = \frac{{1.5y_{6} + 1.9}}{{y_{4} }} - 1 \le 0, \hfill \\ \, g_{11} (y) = \frac{{1.1y_{7} + 1.9}}{{y_{5} }} - 1 \le 0, \hfill \\ \, 2.6 \le y_{1} \le 3.6,0.7 \le y_{2} \le 0.8,17 \le y_{3} \le 28,7.3 \le y_{4} , \hfill \\ y_{5} \le 8.3,2.9 \le y_{6} \le 3.9\;and\;5.0 \le y_{7} \le 5.5 \hfill \\ \end{gathered} \right. \hfill \\ \end{gathered}$$

where, the function *f*(*x*) represents the minimum total cost for minimizing the pressure vessel. The design variables are defined as follows. *x*_1_ is the thickness of the shell; *x*_2_ is the inner radius; *x*_3_ is the length of the cylindrical portion outside the heads. It's important to note that *x*_1_ and *x*_2_ must be multiples of 0.0625 inches, while *x*_3_ is a continuous variable. The optimization problem aims to find the values of these variables that minimize the total cost while satisfying the given constraints.

Table 8 presents the performance comparison results of the WOAAD with other optimization algorithms for the gearbox design problem. Regarding the minimum function value *f*(*x*), the WOAAD obtained an optimization result of = 3169.11*f*(*x*) = 3169.11, which is significantly suitable for the gearbox design problem. Additionally, from the optimization results of other parameters, it is evident that the WOAAD demonstrates stable performance while satisfying the given constraints.

**Table 8:** Performance comparison of different algorithms for speed reducer design problem.

Algorithms	*y*	*y*	*y*	*y*	*y*	*y*	*y*	*f (x)*
WOA	3.5	0.7	17	8.06908	7.91254	3.36744	5.34721	3049.069
GWO	3.50441	0.7	17	8.21166	7.94012	3.3615	5.28857	3013.3366
HHO	3.56467	0.703087	17	8.11144	7.73196	3.72913	5.28665	3150.8959
SSA	3.6	0.7	17	7.75947	8.3	3.9	5.5	3357.4163
WOAAD	3.5	0.7	17	7.59263	8.07378	3.79185	5.34175	3169.11

## Conclusions

This paper introduces a whale optimization algorithm based on atom-like structure differential evolution (WOAAD). It incorporates principles from quantum mechanics, defining the global optimum as the nucleus center and creating concentric circles around it called electron orbits. The algorithm calculates the local optimum within the electron orbit, defining it as the electron orbit's center. During the spiral update phase, it combines the electron orbit center (local optimum) with the original solution using a sine function for coordinated updates. In the contraction encircling phase, the nucleus center (global optimum) is retained for prey encircling. In the random walk foraging phase, both the electron orbit center (local optimum) and random individuals undergo mutation operations, maintaining population diversity while avoiding the randomness of the search. The resulting new individuals are crossed with random dimensions, followed by selection operations to retain better individuals, thus accelerating the algorithm's optimization speed and improving precision. Finally, the algorithm introduces a scout bee strategy. If a whale fails to find a better solution within a specified limit (*L*), it undergoes random initialization to enhance population diversity.

To evaluate the performance of the WOAAD, experiments are conducted using 23 standard benchmark functions, comparing it with other optimization algorithms and other improved WOA. The results demonstrate that the WOAAD significantly improves optimization speed and prevents the algorithm from getting stuck in local optima. Furthermore, the WOAAD is applied to five engineering design problems: cantilever beam, tension spring, three-bar truss, pressure vessel, and gearbox. Experimental results show that the improved algorithm exhibits good applicability (Supplementary Information).

In future research, efforts will be made to enhance the WOAAD performance, conduct experiments using CEC benchmark functions, address multi-objective optimization problems and high-dimensional function optimization problems, and explore applications of the WOAAD in other industrial domains.

### Supplementary Information

Supplementary Information.

## Data Availability

The datasets used and/or analysed during the current study available from the corresponding author on reasonable request.
